# Engineering Morphological Anisotropy to Control the In Vivo Transport Dynamics, Clearance, and Biodistribution of Silica Nanocarriers

**DOI:** 10.1002/advs.77000

**Published:** 2026-08-05

**Authors:** Xiaofei Li, Yingjie Yu, Tianchang He, Xiaoyan Fang, Wenhua Yang, Luying Zhou, Hao Fan, Guimei Lin, Daniel Crespy, Katharina Landfester, Junfeng Zhang, Shuai Jiang

**Affiliations:** ^1^ Key Laboratory of Marine Drugs School of Medicine and Pharmacy Chinese Ministry of Education Ocean University of China Qingdao People's Republic of China; ^2^ Laboratory For Marine Drugs and Bioproducts Qingdao Marine Science and Technology Center Qingdao People's Republic of China; ^3^ School of Pharmaceutical Science Shandong University Jinan People's Republic of China; ^4^ Department of Materials Science and Engineering School of Molecular Science and Engineering Vidyasirimedhi Institute of Science and Technology (VISTEC) Rayong Thailand; ^5^ Max Planck Institute for Polymer Research Mainz Germany

**Keywords:** Core‐shell nanoparticles, drug delivery, liver sequestration, nanomedicines, shape

## Abstract

Shape governs function across scales in nature, from streamlined bacteria to biconcave red blood cells that navigate capillary flow. Inspired by these bio‐geometries, we explore how nanoscale anisotropy can be engineered to control the dynamic transport and biodistribution of synthetic nanocarriers in the body. We fabricate anisotropic silica nanocapsules with precisely tunable asymmetry to dissect shape effects on nano‐bio interactions under physiological flow. Under shear flow in vitro, increasing anisotropy markedly reduced cellular uptake, whereas this effect was much less pronounced under static conditions, revealing strong flow–shape coupling. In vivo, highly anisotropic nanocapsules exhibit prolonged circulation, with a 2.8‐fold longer half‐life than spherical counterparts and significantly reduced sequestration by the liver, spleen, and circulating blood cells. Mechanistic analyses attributed these effects to decreased phagocytic internalization by Kupffer cells, splenic macrophages, and circulating monocytes. Computational fluid dynamics simulations corroborated this phenomenon, demonstrating that greater anisotropy shifts particle trajectories away from vessel walls toward the central flow stream, lowering the chances of cellular interception. Together, these results establish nanoscale anisotropy as a critical determinant of nanoparticle transport, immune recognition, and clearance under flow. Anisotropy engineering therefore provides a nature‐inspired framework for designing long‐circulating, immune‐evasive nanocarriers with improved therapeutic performance.

## Graphical abstract

Morphological anisotropy directs the in vivo transport under physiological flow and clearance of silica nanocarriers. Increasing anisotropy reduces cellular uptake, suppresses hepatic and splenic sequestration, and prolongs systemic circulation by steering particles toward the central flow core. These results highlight particle shape as a key design parameter for long‐circulating, immune‐evasive nanomedicines.

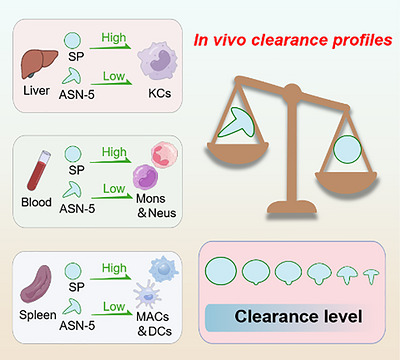

## 1 Introduction

Anisotropy is a universal design strategy in nature that enables living organisms to adapt and function efficiently in dynamic fluidic environments [1, 2, 3, 4]. From the elongated bodies of motile bacteria that enhance propulsion in viscous media to the biconcave morphology of red blood cells that optimizes deformability and flow in capillaries, non‐spherical geometries serve as elegant evolutionary solutions for mechanical efficiency and environmental responsiveness [5, 6, 7]. These biological architectures exemplify how structural anisotropy governs fluid–structure interactions and modulates dynamic behaviors within flowing systems, establishing shape as a key regulator of biological performance.

Translating this principle to the nanoscale presents a new rationale for refining the design of precision nanomedicines. Nanoparticles have revolutionized therapeutic and diagnostic strategies by enabling controlled pharmacokinetics, selective targeting, and reduced systemic toxicity. However, most design efforts have focused on size, charge, and surface chemistry, treating particles largely as isotropic entities [8, 9]. In contrast, nanoparticle anisotropy—the directional dependence of structure and function—remains a relatively underexplored dimension of design, despite its potential to profoundly influence transport, adhesion, and clearance within the body. Early explorations into anisotropic nanoparticles such as rods, disks, stars, and Janus particles have revealed morphology‐dependent differences in cellular uptake and biodistribution [10, 11, 12, 13]. Yet, these studies were typically conducted under static or weakly dynamic conditions, failing to recapitulate the complex hydrodynamic and interfacial forces that nanoparticles experience in vivo. As a result, how shape anisotropy couples with physiological flow to determine nanoparticle fate remains a central and unresolved question in nanomedicine.

**SCHEME 1:**
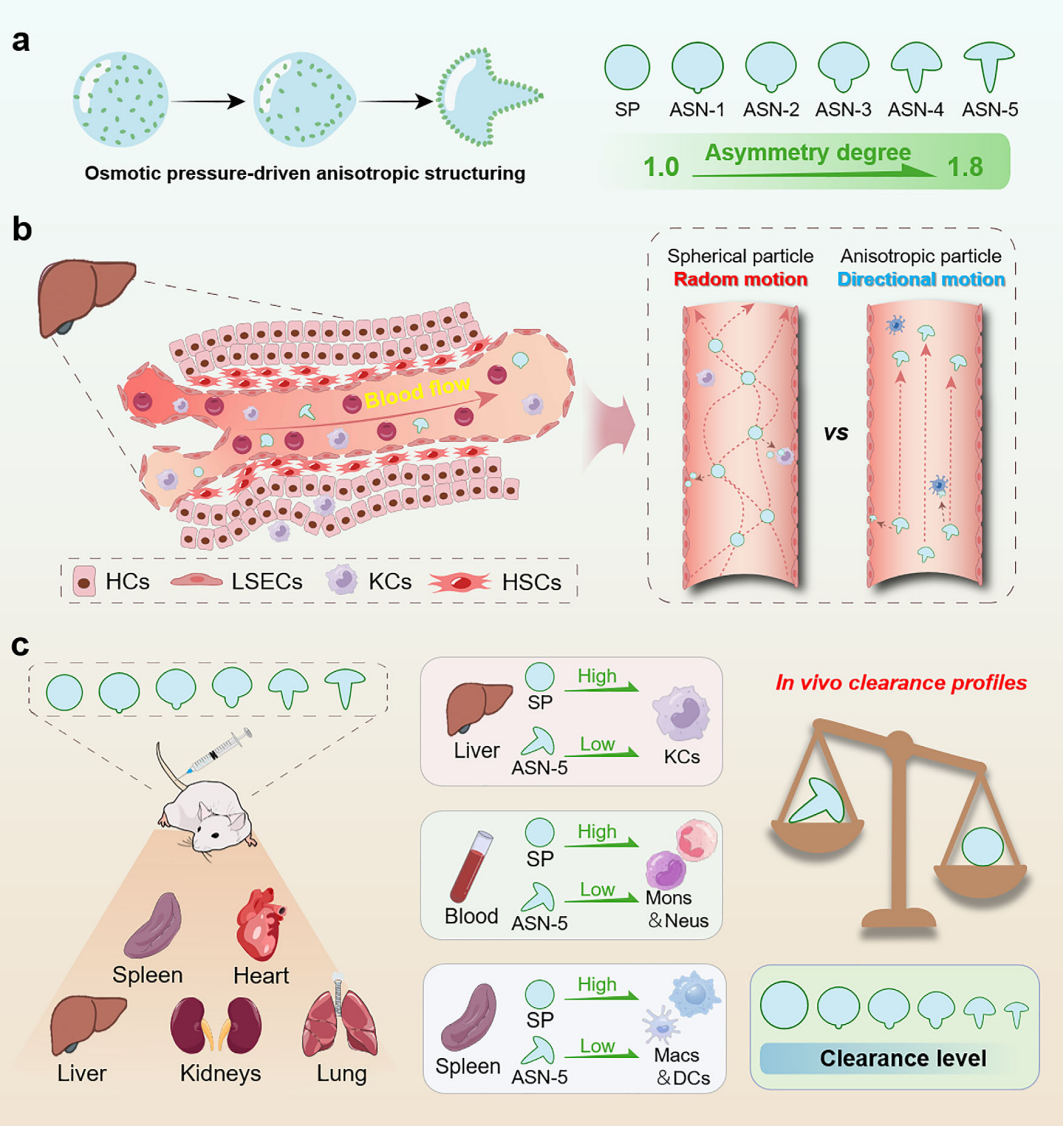
Schematic illustration of shape‐dependent flow behavior and clearance of anisotropic silica nanocapsules (ASNs). (a) Schematic illustration of ASN fabrication. (b) Schematic of shape‐dependent behavior of ASNs in hepatic sinusoids under physiological flow. (c) Biodistribution and clearance mechanisms of ASNs at the sub‐organ level, highlighting differential cellular sequestration in the liver, blood, and spleen.

Herein, we developed anisotropic silica nanocapsules (ASNs) with programmable morphological asymmetry, enabling precise modulation of aspect ratio and curvature (Scheme 1). A family of ASNs with systematically tunable asymmetry (As = 1.0–1.8) were synthesized to elucidate how nanoscale anisotropy modulates nano–bio interactions under physiologically relevant flow. These ASNs, synthesized via a controlled nanoemulsion‐based sol–gel process, exhibited morphologies ranging from “mushroom‐like” to “peg‐like” while maintaining identical size and surface chemistry. Systematic in vitro and in vivo investigations revealed that increasing anisotropy markedly reduced cellular uptake under shear flow, prolonged systemic circulation, and diminished hepatic sequestration. Flow cytometric analysis further revealed distinct cellular tropisms across major organs, with reduced macrophage internalization confirming a shape‐dependent clearance mechanism. Together, these findings establish nanoscale anisotropy as a fundamental determinant of nanocarrier dynamics, biodistribution, and clearance. By bridging fluid dynamics and nanobiology, this work provides a mechanistic framework for understanding anisotropy–fluid–bio coupling and offers guiding principles for the rational design of next‐generation anisotropic nanocarriers with improved circulation and therapeutic performances.

## 2 Results and Discussion

### 2.1 Synthesis of Anisotropic Silica Nanocapsules (ASNs) With Varying Asymmetric Degrees

**FIGURE 1:**
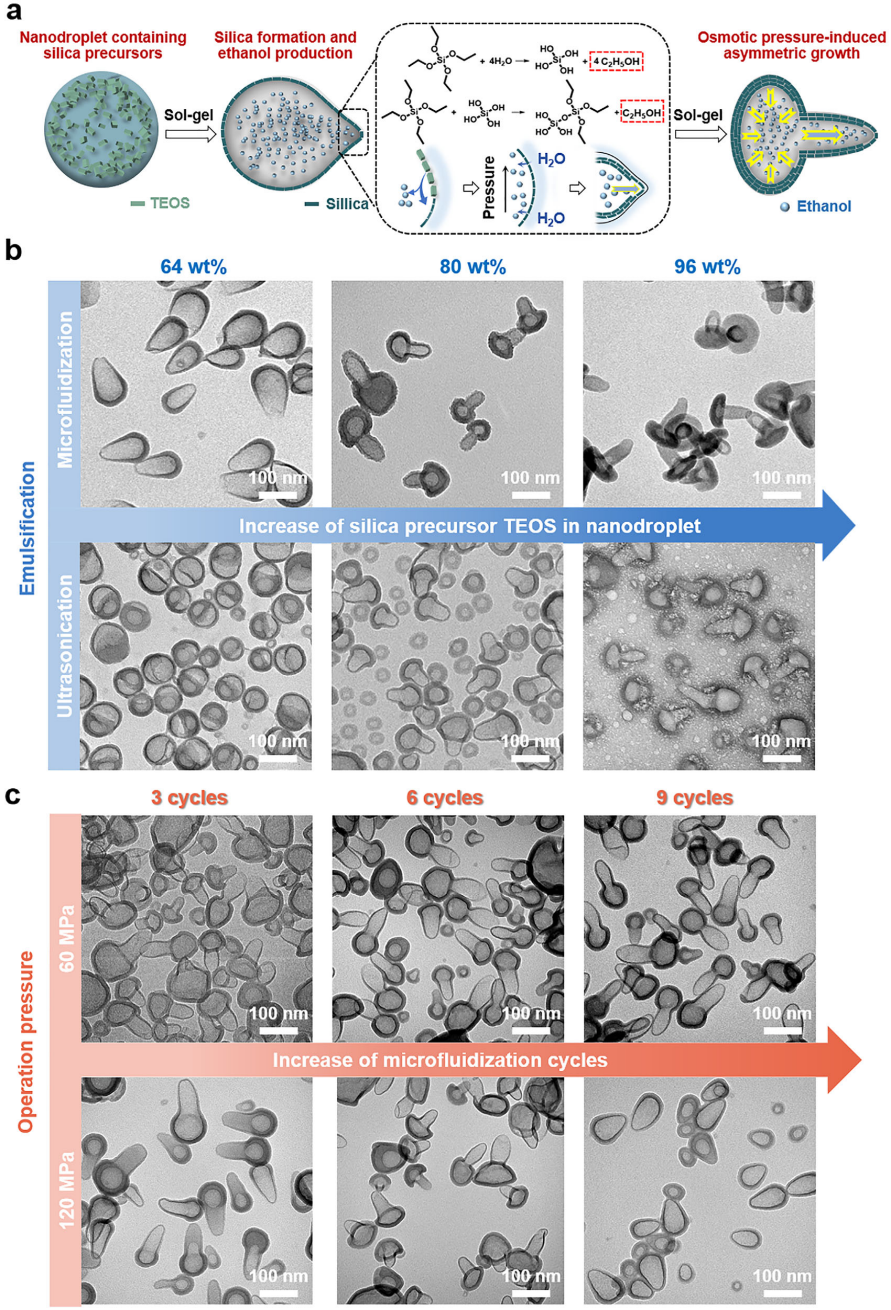
Morphology control of ASNs. (a) Schematic illustration of the formation mechanism of ASNs. (b) TEM images showing the morphological transition of ASNs synthesized with varying TEOS concentrations (64, 80, and 98 wt.%) in the dispersed phase of the emulsion. (c) Effect of microfluidization (MF) parameters on capsule anisotropy for emulsion systems containing 80 wt.% TEOS in the dispersed phase. Horizontal arrow indicates ASNs fabricated with increasing MF cycles (3–9 cycles) under constant pressure. Vertical arrow indicates ASNs prepared under increasing operation pressure (60–120 MPa) at a fixed number of cycles.

The ASNs with a core‐shell architecture were fabricated through a confined sol‐gel reaction of alkoxysilanes at the oil‐water interface in a miniemulsion system [14]. The anisotropic growth of nanocapsules arises from osmotic pressure generated by ethanol during hydrolysis and condensation of tetraethyl orthosilicate (TEOS). During emulsification, TEOS undergoes hydrolysis upon contact with water at the droplet interface, producing ethanol as a byproduct. The formation of an initial silica shell fixes droplet size, while continuous hydrolysis within the droplet gradually replaces the TEOS core with ethanol. The resulting ethanol concentration gradient between the droplet interior and the aqueous phase drives osmotic water influx, forcing the hydrophobic interior outward and inducing anisotropic deformation of the forming capsule (Figure 1a) [15].

Systematic variation of precursor composition in the dispersed phase revealed that morphology is highly sensitive to the TEOS‐to‐chloroform ratio. Increasing TEOS content from 64 to 80 wt.% led to the formation of asymmetric “tank–tail” or “mushroom‐like” structures, whereas further elevation to 98 wt.% yielded highly elongated nanonail morphologies (Figure 1b). As TEOS concentration increased, nanocapsules exhibited a gradual morphological transformation from spherical to highly anisotropic forms. Notably, similar trends were observed when emulsification was performed using ultrasonication, confirming that anisotropy originates essentially from TEOS‐driven osmotic imbalance rather than emulsification modality.

We further examined how processing parameters influence anisotropic formation. At low microfluidization pressure (60 MPa), only mild deformation was observed irrespective of TEOS content (80 or 98 wt.%). In contrast, higher pressure (120 MPa) markedly enhanced shape anisotropy, yielding more pronounced deformations (Figure 1c, Figure S1). Moreover, increasing the number of microfluidization cycles further promoted anisotropic evolution, highlighting the effect of shear stress in directing particle deformation. Together, these findings underscore the synergistic roles of precursor concentration, osmotic pressure, and shear stress in directing non‐equilibrium shape evolution.

**FIGURE 2:**
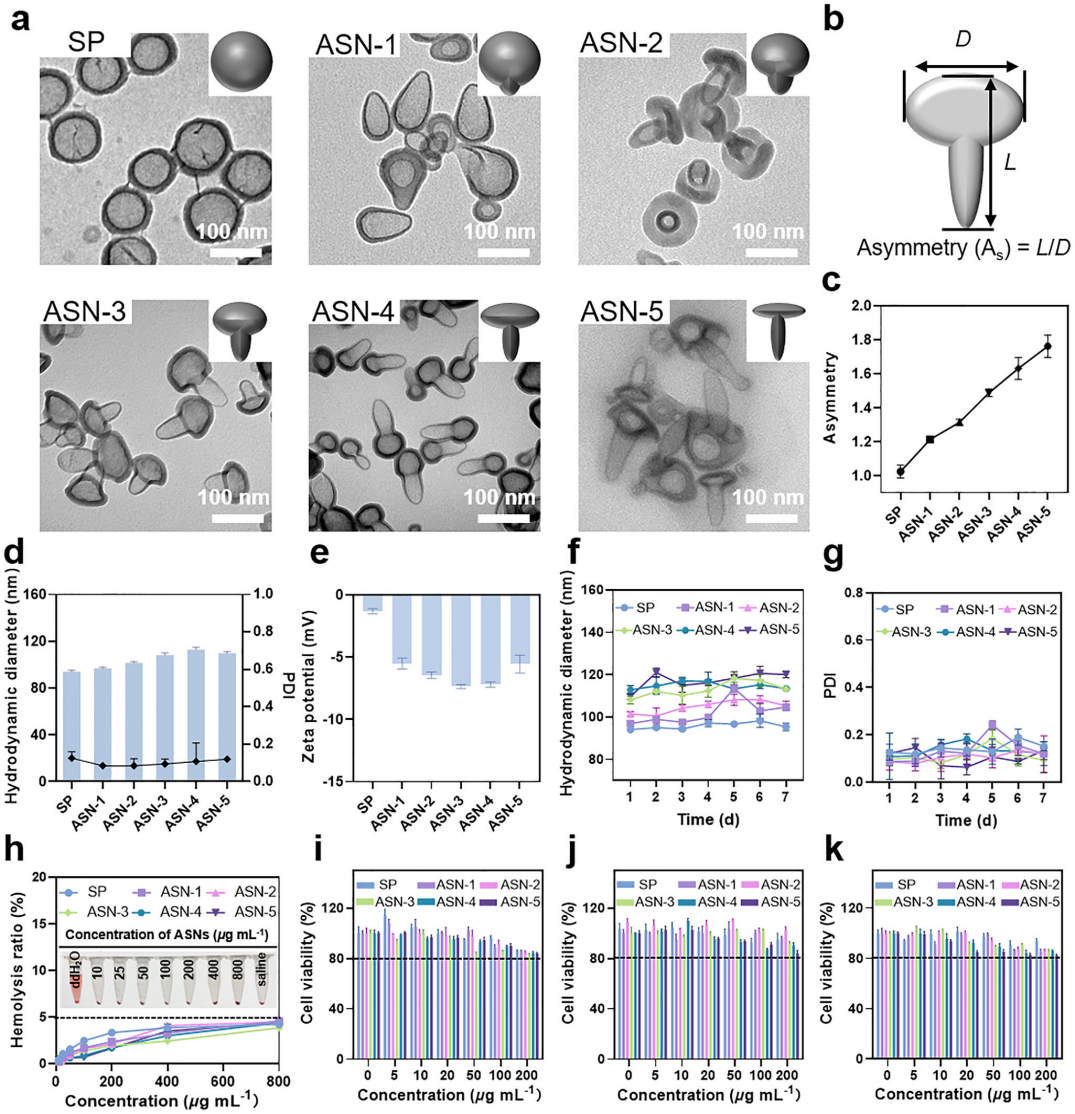
Physiochemical characterization and biocompatibility assessment of ASNs with varying morphological anisotropy. (a) TEM images of ASNs exhibiting increasing anisotropy (SP to ASN‐5). The formulations shown in Figure 2a were selected from the processing‐parameter optimization experiments shown in Figure 1c and Figure S1. The corresponding samples are as follows: SP: corresponding to the sample in Figure 1b prepared with 64 wt.% TEOS under ultrasonication at 70% amplitude; ASN‐1: corresponding to the sample in Figure 1c prepared with 80 wt.% TEOS under microfluidization at 120 MPa for 9 cycles; ASN‐2: corresponding to the sample in Figure S1 prepared with 96 wt.% TEOS under microfluidization at 60 MPa for 9 cycles; ASN‐3: corresponding to the sample in Figure 1c prepared with 80 wt.% TEOS under microfluidization at 120 MPa for 6 cycles; ASN‐4: corresponding to the sample in Figure 1c prepared with 80 wt.% TEOS under microfluidization at 60 MPa for 9 cycles; ASN‐5: corresponding to the sample in Figure S1 prepared with 96 wt.% TEOS under microfluidization at 120 MPa for 9 cycles. Scale bar: 100 nm. (b) Schematic illustration of ASNs showing the geometric parameters used to define asymmetry. The asymmetry index (A_s_) was calculated as A_s_ = *L*/*D*, where *L* denotes the total longitudinal length (head + tail) and *D* represents the head diameter. (c) Quantitative analysis of ASN asymmetry, based on measurements from at least 50 nanocapsules per group from TEM images using Nano Measurer. (d) Hydrodynamic diameter and (e) zeta potential of ASNs. (f) Hydrodynamic diameter and (g) PDI of ASNs monitored over a 7‐day storage period in PBS (*n* = 3). (h) Hemolysis of ASNs at various concentrations (*n* = 3). The inset shows corresponding hemolysis images, with the dashed line marking the 5% safety threshold. (i‐k) Cell viability of HUVEC (i), L929 (j), and RAW264.7 (k) cells treated with ASNs at various concentrations. Data are presented as mean ± SD (*n* = 3).

Based on the optimized fabrication parameters, we constructed a library of shape‐engineered nanocapsules, including spherical (SP) and five anisotropic variants (ASN‐1 to ASN‐5) with progressively increased morphological asymmetry (Figure 2a). The degree of anisotropy was quantified using an asymmetry index (A_s_ = *L*/*D*), where *L* denotes the total longitudinal length (head + tail) and *D* represents the head diameter (Figure 2b). The index increased from 1.0 (SP) to 1.8 (ASN‐5), establishing a well‐defined gradient of shape anisotropy (Figure 2c). Despite the morphological divergence, the hydrodynamic diameters (∼ 100 nm) and surface charge (0 ∼ −10 mV) were remained identical among the groups (Figure 2d,e). All formulations exhibited good colloidal stability during storage at 4°C for seven days, maintaining polydispersity index (PDI) below 0.3 and showing negligible size fluctuations (Figure 2f,g).

To ensure biosafety, we conducted hemolysis and in vitro cytocompatibility assessments. All nanocapsules induced < 5% hemolysis, with no visible erythrocyte disruption, confirming favorable hemocompatibility (Figure 2h). Moreover, cell viability in HUVECs, L929 fibroblasts, and RAW264.7 macrophages remained above 80% at nanocapsule concentrations up to 200 µg mL^−^^1^, indicating that morphology tuning does not compromise the biocompatibility of the ASNs (Figure 2i–k).

### 2.2 Pharmacokinetics and Biodistribution of Anisotropic Nanocapsules

**FIGURE 3:**
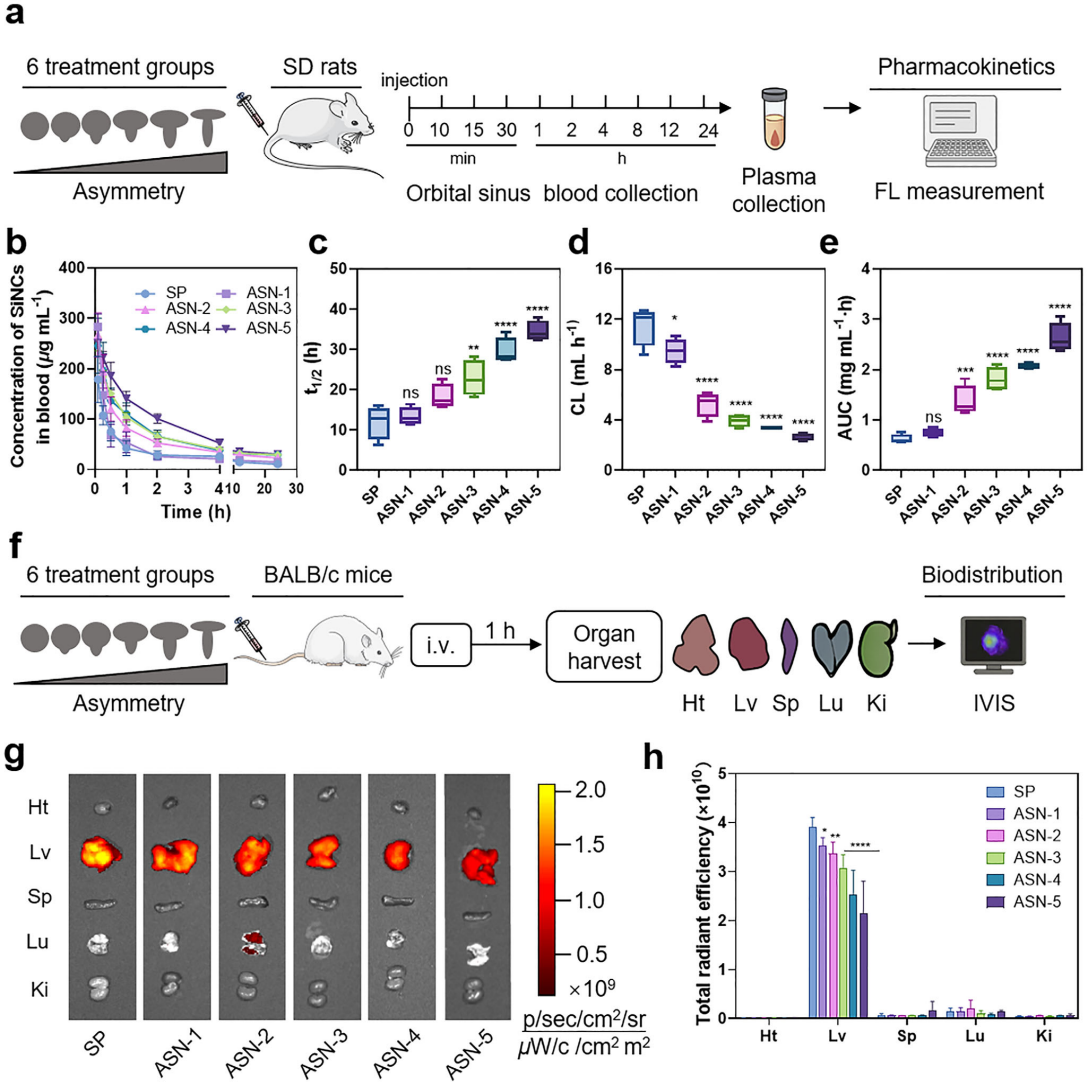
In vivo circulation and organ distribution of the ASNs. (a) Schematic illustrating the pharmacokinetic study design (*n* = 4). (b) Blood concentration‐time curves of ASNs. (c) Plasma half‐life (t_1/2_). (d) Clearance rate. (e) Area under the blood concentration‐time curve (AUC). (f) Schematic illustrating the biodistribution study workflow (*n* = 3). (g) Ex vivo fluorescence images of major organs collected 1 h post‐injection. (h) Quantification of organ fluorescence using Living Image software. Data are presented as mean ± SD. Statistical significance was analyzed using one‐way ANOVA followed by Tukey's multiple‐comparison test. For anisotropy‐related comparisons, SP was used as the reference group, with *p* < 0.05 considered statistically significant. *, **, ***, and **** represent *p* < 0.05, *p* < 0.01, *p* < 0.001, and *p* < 0.0001, respectively.

To investigate the in vivo clearance behavior of ASNs, pharmacokinetic studies were performed in SD rats (Figure 3a). Fluorescence spectra confirmed the successful fluorescent labeling of ASNs with Cy5 (Figure S2). As shown in Figure 3b, all formulations exhibited a rapid initial distribution phase within the first hour after intravenous injection, followed by a slower elimination phase after approximately 4 h, indicative of a typical biphasic pharmacokinetic profile. Notably, compared with SP, ASNs—particularly ASN‐4 and ASN‐5—displayed a markedly prolonged elimination phase and sustained plasma exposure at later time points, indicating that increased particle anisotropy effectively retards systemic clearance and prolongs blood circulation. Pharmacokinetic parameters were calculated using a standard two‐compartment model in PKSolver (Figure 3c–e). This model was used because the blood fluorescence–time profiles showed a biphasic decline, consisting of an initial rapid distribution phase followed by a slower elimination phase, which is consistent with nanoparticle distribution between central and peripheral compartments. The model assumes instantaneous mixing within each compartment and first‐order elimination kinetics. The fitted curves showed good agreement with the experimental data, with R^2^ values greater than 0.99 for all groups. Additional diagnostic parameters, including WSS, SE, AIC, and SC, are summarized in Table S1. R^2^ was used to assess the agreement between fitted and experimental values, while WSS, SE, AIC, and SC were reported as additional diagnostic parameters supporting that the two‐compartment model adequately fitted the experimental data. Notably, the circulation half‐life (t_1_/_2_) increased substantially with morphological anisotropy. ASN‐5 displayed a t_1_/_2_ of ∼34 h, approximately 2.8‐fold longer than that of spherical nanocapsules (SP, ∼12 h), indicating markedly prolonged systemic retention. Correspondingly, the clearance rate (CL) decreased progressively from 11.5 mL·h^−^^1^ (SP) to 2.7 mL·h^−^^1^ (ASN‐5), representing an approximate 4.2‐fold reduction. The area under the curve (AUC) showed similar anisotropy‐dependent enhancement: ASN‐5 achieved an AUC of ∼2.63 mg·mL^−^^1^·h, which is ∼3.5‐fold higher than that of SP (∼0.75 mg·mL^−^^1^·h). Collectively, these results demonstrate that increasing morphological anisotropy significantly attenuates systemic clearance and prolongs the in vivo circulation lifetime of ASNs.

We further evaluated how anisotropy influences the in vivo biodistribution of ASNs in BALB/c mice (Figure 3f). Whole‐body and ex vivo imaging were performed 1 h post‐injection to evaluate accumulation in major organs (heart, liver, spleen, lungs, and kidneys) as well as in circulating blood (Figure 3g,h and Figure S3). As shown in Figure 3g, all ASNs exhibited predominant liver accumulation with negligible signals in other organs, consistent with the well‐recognized role of the reticuloendothelial system (RES), where Kupffer cells dominate nanoparticle sequestration, with additional contributions from splenic and bone‐marrow macrophages [16, 17, 18, 19].

Importantly, liver accumulation exhibited a clear anisotropy‐dependent decline (Figure 3h). Relative to SP, hepatic fluorescence intensity decreased by 10.2%, 15.0%, 20.8%, 31.5%, and 41.6% for ASN‐1 through ASN‐5, respectively, indicating that higher anisotropy markedly reduces liver retention. Consistently, fluorescence analysis of the blood showed that ASN‐5 maintained substantially higher levels than the other groups (Figure S3). Together, these results demonstrate that increasing morphological anisotropy suppresses hepatic uptake while enhancing systemic retention, establishing anisotropy as an effective design parameter for modulating the in vivo fate of nanomedicines. All ASNs used for in vivo evaluation were PEGylated using the same procedure to improve colloidal stability and maintain comparable surface chemistry. Accordingly, the biological differences observed here should be interpreted as shape‐dependent responses under PEGylated conditions, rather than as intrinsic shape effects independent of surface modification. Future studies directly comparing PEGylated and non‐PEGylated ASNs will help clarify how PEGylation interacts with particle anisotropy to influence biological behavior.

### 2.3 Cellular Uptake of ASNs Under Static and Dynamic Flow Conditions

**FIGURE 4:**
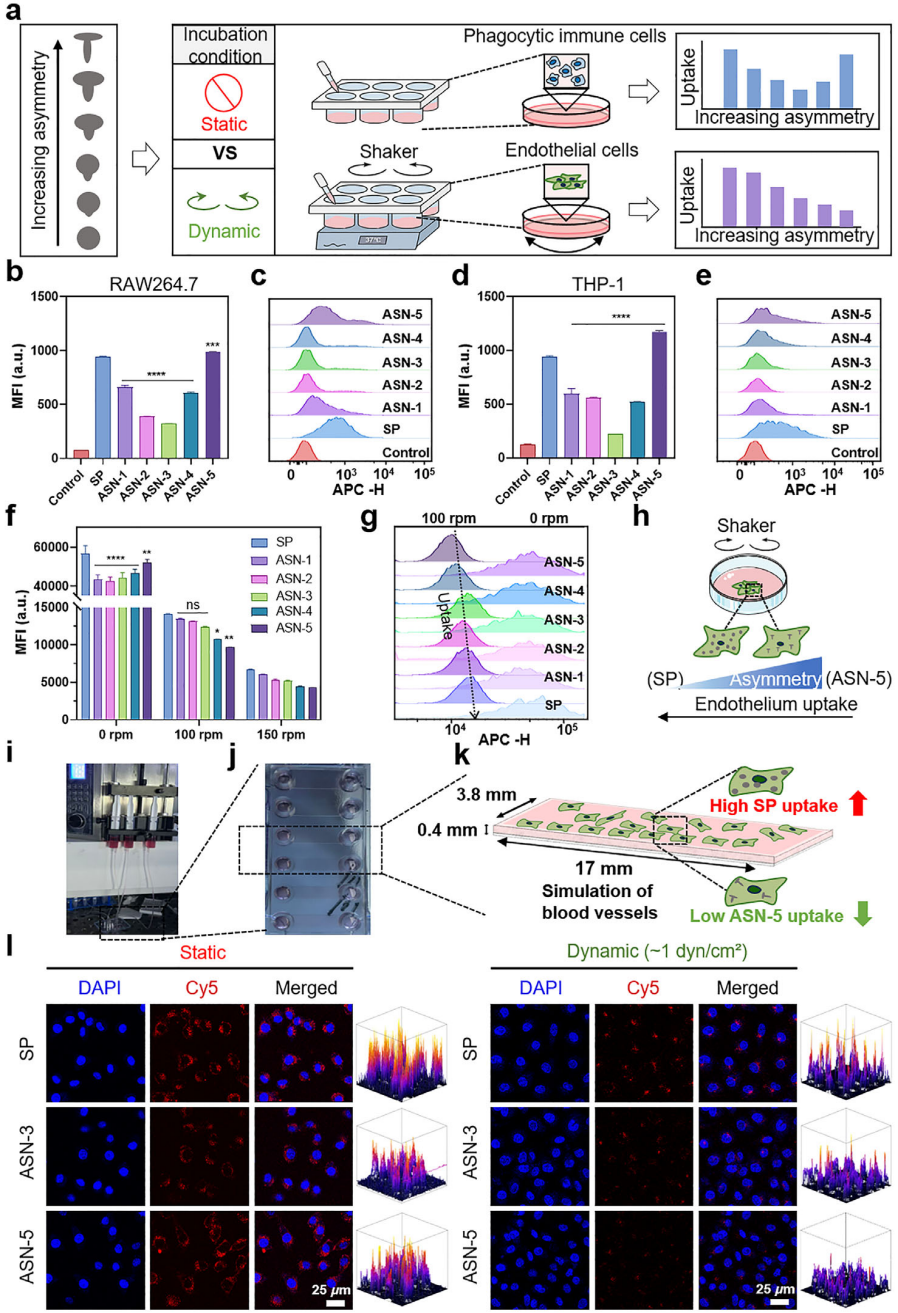
In vitro static and dynamic simulation of cellular uptake of ASNs. (a) Schematic illustration of the workflow for evaluating cellular uptake under static (0 rpm) and dynamic (shaker‐induced flow) conditions. (b‐e) MFI and corresponding histogram profiles showing the uptake of ASNs by RAW264.7 and THP‐1 cells after 1 h incubation. (f) MFI of internalized ASNs under increasing flow rates (0, 100, and 150  rpm) and (g) representative flow cytometry histograms under static (0 rpm) and dynamic (100  rpm) conditions. (h) Schematic representation of how ASN asymmetry modulates endothelial internalization under shear flow conditions. (i) Photograph of the peristaltic‐pump‐assisted perfusion system. (j) Photograph of the µ‐Slide VI 0.4 flow chamber. (k) Schematic illustration of the HUVEC‐lined microchannel model under flow. (l) CLSM images of HUVECs incubated with Cy5‐labeled SP, ASN‐3, and ASN‐5 in the microfluidic system under static or dynamic flow conditions. Dynamic flow was applied at a laminar shear stress of ∼1 dyn cm^−^^2^. Cell nuclei were stained with DAPI, and nanoparticles were visualized by Cy5 fluorescence. Fluorescence intensity profiles of Cy5 signals are shown on the right. Scale bar: 25 µm. RPMIStatistical significance was analyzed using one‐way ANOVA followed by Tukey's multiple‐comparison test. For anisotropy‐related comparisons, SP was used as the reference group, with *p* < 0.05 considered statistically significant. *, **, ***, and **** represent *p* < 0.05, *p* < 0.01, *p* < 0.001, and *p* < 0.0001, respectively.

The mononuclear phagocyte system (MPS), comprising circulating monocytes, tissue‐resident macrophages, and related antigen‐presenting cells, plays a central role in the recognition and clearance of circulating particulates [20, 21]. To examine how anisotropic ASNs interact with immune cells, in vitro uptake studies were performed using RAW264.7 and THP‐1 macrophages (Figure 4a). All ASN formulations exhibited comparable fluorescence‐labeling efficiencies and similar sedimentation behaviors (Figures S4 and S5), thereby excluding potential artifacts in uptake measurements arising from differences in labeling intensity or gravitational settling. To elucidate the internalization pathways, cellular uptake was assessed in the presence of pathway‐specific inhibitors. The results showed that ASN uptake was predominantly energy‐dependent and mediated by macropinocytosis, with no significant differences in inhibitor sensitivity across different particle morphologies (Figure S6). These results indicate that morphological anisotropy does not alter the fundamental uptake mechanism.

Interestingly, mean fluorescence intensity (MFI) analysis revealed a nonlinear dependence of cellular uptake on particle asymmetry (Figure 4b‐e). Both RAW264.7 and THP‐1 macrophages exhibited minimal uptake of ASNs with intermediate asymmetry (ASN‐2, ASN‐3), whereas particles with low‐anisotropy (ASN‐1) or high elongation (ASN‐5) showed enhanced internalization. This trend is consistent with previous reports demonstrating suppressed macrophage uptake of nanocarriers with intermediate asymmetry relative to spherical or highly elongated structures [13]. A theoretical model proposed by Dasgupta et al. suggests that ellipsoidal nanocapsules with moderate aspect ratios tend to adopt stable, partially wrapped membrane configurations that hinder complete engulfment [22]. Recent reviews further attribute this phenomenon to the coupled effects of membrane contact area, strain energy, and particle orientation at the bio–nano interface [23]. Together, these findings show the cellular uptake profiles of different anisotropic nanocarriers under static culture conditions, where cellular interactions occur in the absence of hemodynamic forces. In vivo, however, circulating nanocarriers encounter immune and endothelial cells under continuous shear stress and dynamic flow.

To evaluate ASN uptake under more physiologically relevant conditions, human umbilical vein endothelial cells (HUVECs) were incubated with fluorescently labeled ASNs under static (0 rpm) and dynamic flow conditions (100 and 150 rpm, induced using an orbital shaker) (Figure 4a). The shear stresses generated by the orbital‐shaker system were estimated from the operating parameters, including culture‐plate geometry, medium volume, viscosity, orbital radius, and rotation speed, according to previously reported hydrodynamic analyses of orbital culture systems [24, 25, 26]. The estimated shear stresses were approximately 0.15–0.35 Pa at 100 rpm and 0.40–0.90 Pa at 150 rpm, which fall within the low‐to‐moderate shear regime reported for physiological microcirculatory environments (Table S2). For comparison, shear stresses in hepatic sinusoids are typically in the range of 0.01–0.5 Pa, whereas capillaries and post‐capillary venules generally experience shear stresses of approximately 0.1–2 Pa. In contrast, larger arteries are exposed to substantially higher shear stresses, typically ranging from 1 to 7 Pa. Therefore, the dynamic conditions employed in this study are more representative of low‐shear microvascular and sinusoidal environments than of high‐shear arterial flow. Under static incubation, MFI exhibited a biphasic dependence on asymmetry, decreasing for intermediate asymmetry and increasing again for highly anisotropic ASNs (Figure 4f,g). In contrast, under dynamic flow, cellular uptake decreased monotonically with increasing anisotropy, with the strongest suppression observed at 150 rpm, where uptake was reduced to ∼ 10% of the static level. These results are consistent with previous reports showing that shear flow suppresses nanocarrier internalization by endothelial cells [27]. Flow‐induced alignment of anisotropic particles along the streamlines reduces their effective rotational diffusion and residence time in the near‐wall region, thereby decreasing collision probability with cell membrane. Moreover, shear stress can induce subtle alterations in membrane fluidity and cytoskeletal tension, potentially impairing endocytic initiation and maturation, which collectively contribute to the reduced nanoparticle uptake [28, 29]. Under flow conditions, anisotropic ASNs exhibited a pronounced reduction in cellular uptake, and this effect became more pronounced with increasing anisotropy (Figure 4h). Confocal laser scanning microscopy corroborated these findings (Figure S7), showing strong perinuclear Cy5 fluorescence (red) under static conditions but markedly diminished signals under flow, especially for ASN‐5. Consistent with prior experimental and computational studies, particle geometry critically influences margination behavior and near‐wall residence time; elongated or asymmetric nanoparticles are less prone to sustained wall contact and adhesion, thereby reducing their probability of internalization under shear flow [4, 30]. Taken together, these results demonstrate that flow dynamics regulate endothelial interactions in an asymmetry‐dependent manner, underscoring the pivotal role of particle anisotropy in determining cellular recognition under physiological circulation conditions.

To further evaluate nanoparticle behavior under physiologically relevant flow conditions, a HUVEC‐lined microfluidic platform was established to provide a controllable endothelial flow interface under laminar shear stress (∼1 dyn cm^−^^2^), partially recapitulating the hemodynamic environment of hepatic microcirculation (Figure 4i–k). Cy5‐labeled SP, ASN‐3, and ASN‐5 were introduced into the microchannels, and their cellular association was examined by confocal microscopy. The microfluidic results showed a trend similar to that observed in the orbital‐shaker model. Under static conditions, SP and ASN‐5 showed relatively stronger Cy5 signals, whereas ASN‐3 displayed the weakest cellular association. In contrast, under flow conditions, the shape‐dependent difference became more evident, with highly anisotropic ASN‐5 showing reduced endothelial association compared with SP (Figure 4l). These results suggest that nanoparticle anisotropy affects cell–particle interactions more clearly under flow than under static incubation.

This flow‐dependent reduction in cellular association is consistent with the orbital‐shaker experiments, and in vivo biodistribution results. Highly anisotropic nanoparticles may undergo altered rotational and hydrodynamic behavior under flow, reducing their tendency to approach or remain near the endothelial surface. Such reduced wall‐associated interaction may contribute to lower hepatic accumulation and decreased uptake by phagocytic cells in vivo. Although the HUVEC‐based microfluidic model does not reproduce the specialized features of LSECs or the full complexity of blood hepatic sinusoidal microenvironment, including LSECs, fenestrations, and Kupffer cells, it provides a more controlled flow environment than orbital shaking alone and further supports the role of anisotropy in regulating nanoparticle behavior under dynamic flow conditions.

### 2.4 Computational Fluid Dynamics (CFD) Simulation of ASNs in Blood Vessels

**FIGURE 5:**
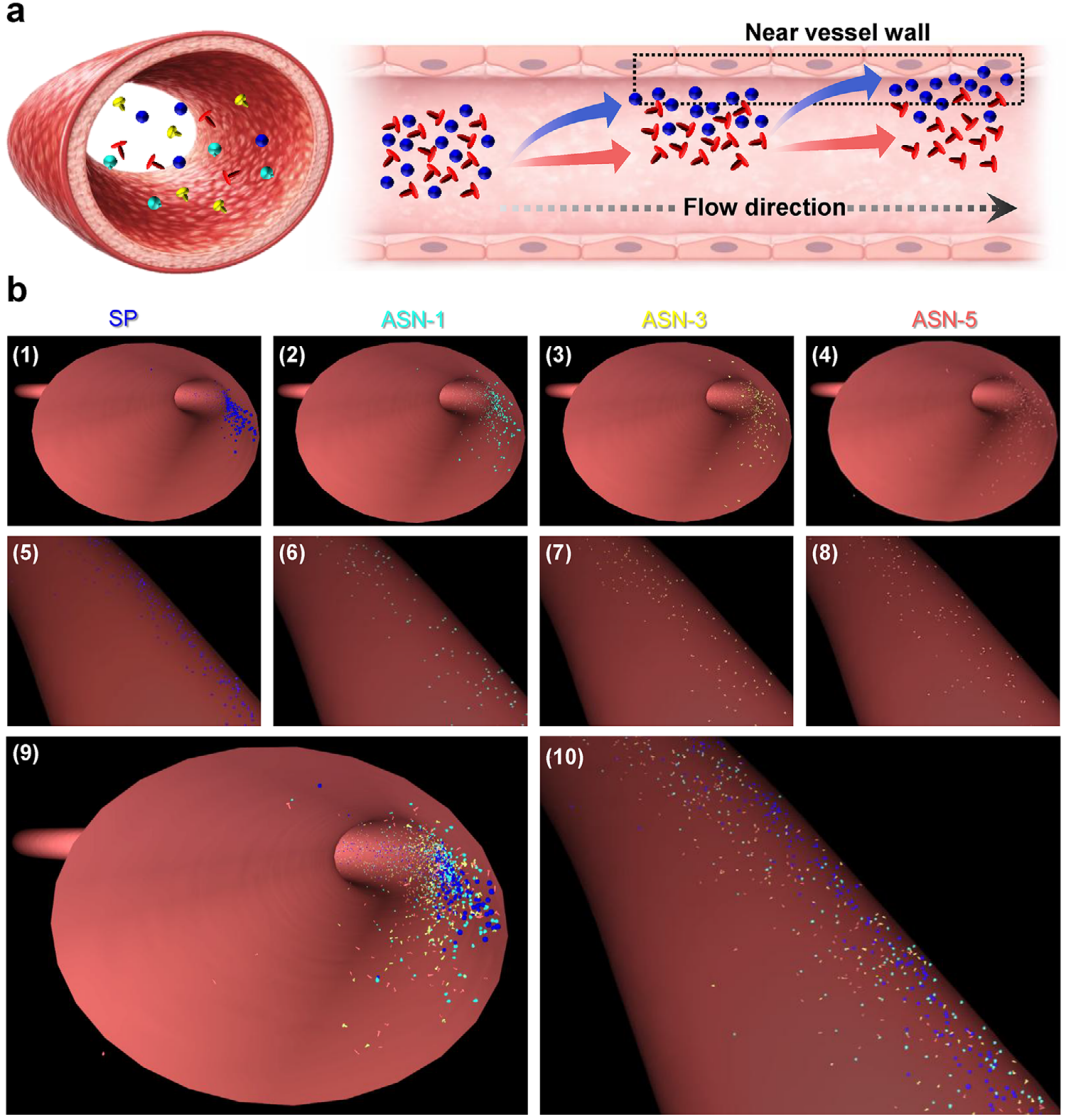
CFD simulation of ASNs in blood vessels. (a) Schematic illustration and representative cross‐sectional views of ASNs flowing through a cylindrical blood vessel, illustrating the radial distribution of particles within the vessel lumen. (b) Simulation snapshots shown from different perspectives: (1–4) cross‐sectional views of SP, ASN‐1, ASN‐3, and ASN‐5; (5–8) corresponding top‐view (axial projection) images; (9–10) merged views combining both perspectives.

Computational modeling and fluid dynamics simulations were performed to systematically investigate the transport behavior of ASNs and their interactions with the vascular wall under flow conditions (Figure 5a). Three‐dimensional CFD simulations were conducted in a mildly curved vessel geometry that mimics hepatic sinusoidal vasculature (Figure S8), allowing quantitative analysis of the spatial distributions of silica nanocapsules with varying degrees of morphological anisotropy. This modeling framework enabled elucidation of how nanoscale shape anisotropy modulates blood‐flow‐mediated transport and wall interactions of particles. Shape‐dependent transport dynamics and wall interactions under shear flow visualized using CFD simulations were shown as Video S1.

As shown in Figure 5b, SP exhibited pronounced margination behavior, with a high probability of localizing near the vessel wall under flow. In contrast, increasing morphological anisotropy progressively altered the radial distribution of nanoparticles. Low‐anisotropy particles (ASN‐1) displayed a modest decrease in near‐wall probability, whereas highly anisotropic particles (ASN‐3 and ASN‐5) preferentially remained in the central flow region, exhibiting markedly reduced proximity to the vessel wall.

This trend is consistent with previous reports demonstrating that nanoparticle shape plays a critical role in regulating vascular transport under flow [31]. Spherical particles, owing to their geometric symmetry, experience more isotropic hydrodynamic forces, which favor frequent lateral migration and wall accumulation. In contrast, anisotropic particles are subjected to asymmetric hydrodynamic torques and orientation‐dependent drag forces, which suppress margination and reduce wall encounters. Such altered flow–particle interactions are expected to promote prolonged circulation and mitigate nonspecific vascular wall adhesion and organ sequestration.

It should be noted that the CFD model was designed as a simplified microvascular flow system relevant to hepatic circulation, rather than a biomimetic liver sinusoid model. Thus, key sinusoidal features, such as low shear flow, fenestrated endothelium, intraluminal Kupffer cells, and direct nanoparticle–cell interactions, were not explicitly included. In addition, the model treated blood as a homogeneous fluid and therefore did not account for the presence of erythrocytes or erythrocyte‐induced hydrodynamic effects, including particle margination, collision‐induced diffusion, and cell‐free layer formation. Within this simplified framework, highly anisotropic nanoparticles remained closer to the central streamlines and showed less migration toward the vessel wall than more symmetric particles. This reduced wall‐approach tendency may decrease the probability of particle contact with endothelial cells and wall‐associated phagocytic cells under flow. Therefore, the CFD results are interpreted as supportive hydrodynamic evidence for morphology‐dependent transport behavior, rather than as a direct simulation of hepatic sinusoidal clearance.

### 2.5 Clearance Mechanisms of Anisotropic Nanocarriers In Vivo

**FIGURE 6:**
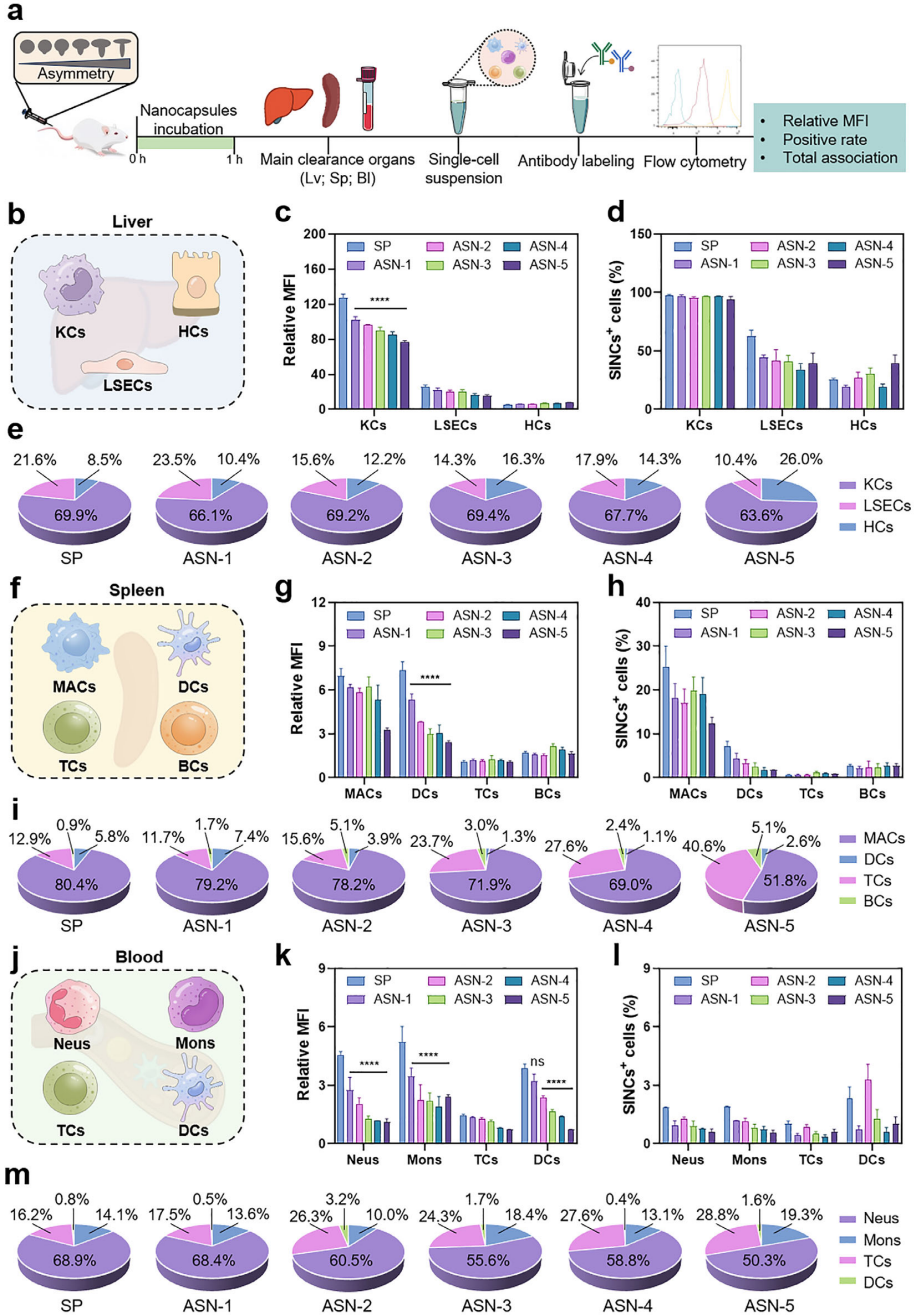
Cell tropism of ASNs in the liver, spleen and blood. (a) Schematic illustration of the workflow for isolating distinct cell populations from blood, liver, and spleen 1 h after intravenous administration of ASNs. (b) Schematic representation of hepatic cell populations: Kupffer cells (KCs), liver sinusoidal endothelial cells (LSECs), and hepatocyte‐enriched cells, referred to as HCs for simplicity. (c) MFI of internalized ASNs in hepatic cell subsets (*n =* 3). (d) Percentage of ASN‐positive hepatic cells (*n =* 3). (e) Contribution of each hepatic cell type to total hepatic uptake of ASNs. (*n =* 3). (f) Schematic diagram of splenic cell populations: macrophages (MACs), dendritic cells (DCs), T cells, and B cells. (g) MFI of ASNs in splenic cell subsets (*n =* 3). (h) Percentage of ASN‐positive splenic cells (*n =* 3). (i) Contribution of each splenic cell type to total splenic uptake of ASNs. (j) Schematic diagram of major blood cell populations: monocytes, neutrophils, T cells, and B cells. (k) MFI of ASNs in blood cell subsets (*n =* 3). (l) Percentage of ASN‐positive blood cells (*n =* 3). (m) Contribution of each blood cell type to total sequestration in circulation (*n =* 3). Statistical significance was analyzed using one‐way ANOVA followed by Tukey's multiple‐comparison test. For anisotropy‐related comparisons, SP was used as the reference group, with *p* < 0.05 considered statistically significant. *, **, ***, and **** represent *p* < 0.05, *p* < 0.01, *p* < 0.001, and *p* < 0.0001, respectively.

Liver, spleen, and blood constitute the major reticuloendothelial compartments responsible for nanoparticle clearance [4, 32, 33, 34]. The liver serves as the primary sink for circulating particles, mainly through uptake by Kupffer cells (KCs) and liver sinusoidal endothelial cells (LSECs), whereas the spleen acts as a secondary filtration organ enriched in macrophages and dendritic cells. In parallel, circulating immune cells in the blood can actively scavenge nanoparticles before they reach peripheral tissues. Elucidating how particle anisotropy modulates these interactions is therefore essential for understanding the in vivo clearance behavior of nanoparticles. Our pharmacokinetic and biodistribution results revealed that ASN anisotropy profoundly affects tissue retention and systemic circulation. To further dissect the cellular mechanisms underlying these observations, we systematically quantified the uptake of ASNs with varying aspect ratios (A_s_) by cells in the liver, spleen, and blood (Figure 6a).

Liver tissues were harvested 1 h post‐injection, and isolated hepatic cells were labeled by fluorophore‐conjugated antibodies (KCs: FITC F4/80; LSECs: PE CD146) and analyzed by flow cytometry. Uptake was quantified across three major hepatic cell populations: KCs, LSECs, and hepatocyte‐enriched cells, hereafter referred to as HCs for simplicity. (Figure 6b, Figure S9). As shown in Figure 6c–e, KCs exhibited substantially higher uptake than LSECs and HCs. Notably, the MFI in KCs decreased progressively with increasing particle anisotropy, with the highly elongated ASN‐5 exhibiting nearly a twofold lower uptake compared with SP. These results indicate that higher anisotropy significantly suppresses macrophage‐mediated phagocytosis in the liver. Relative contribution analysis (Figure 6e) further showed that although per‐cell uptake decreased at higher anisotropy, KCs remained the dominant contributors to hepatic clearance, accounting for approximately 68% of total liver uptake.

Spleen is a key immune filtration organ enriched in phagocytic and antigen‐presenting cells [35, 36]. Among its cellular constituents, macrophages (MACs) and dendritic cells (DCs) are primarily responsible for nanoparticle sequestration, whereas T and B lymphocytes mainly participate in adaptive immune regulation [37, 38]. Analysis of these subsets thus helps elucidate how particle anisotropy influences splenic clearance behavior. Flow cytometry results showed that splenic MACs exhibited the most pronounced reduction in ASN uptake with increasing anisotropy, while DCs showed a more moderate decline. In contrast, T and B cells consistently displayed minimal uptake regardless of particle shape (Figure 6f–h, Figure S10). Relative contribution analysis confirmed that splenic MACs dominated ASN sequestration (Figure 6i). Although overall uptake by splenic MACs was lower than that of hepatic KCs, both populations exhibited a similar anisotropy‐dependent reduction, confirming macrophage‐mediated clearance as the dominant pathway for ASN elimination from circulation.

In the blood, neutrophils (Neus) and monocytes (Mons) showed the highest uptake efficiency, whereas lymphocytes exhibited minimal association with ASNs (Figure 6j–m, Figure S11). Low‐anisotropy particles (SP, ASN‐1) were internalized more readily by Neus and Mons, whereas high‐anisotropy particles (ASN‐3 to ASN‐5) demonstrated markedly reduced cellular association. These trends align with observations in the liver and spleen, indicating that increased anisotropy enables nanoparticles to evade uptake by both tissue‐resident and circulating phagocytes. These results demonstrate that increasing morphological anisotropy confers enhanced stealth properties to ASNs across the reticuloendothelial system, thereby reducing clearance by immune cells and prolonging systemic circulation. It should be noted that, because CD45 pre‐gating was not included in the flow‐cytometry analysis, the uptake data should be interpreted as ASN‐associated fluorescence within these marker‐defined, phagocyte‐enriched populations, rather than definitive immune‐cell subsets.

The relationships between nanoparticle asymmetry and biological behavior were analyzed between the A_s_ and several representative in vivo parameters, including circulation half‐life, clearance rate, AUC, liver accumulation, Kupffer‐cell uptake, splenic macrophage uptake, and circulating monocyte/neutrophil uptake. The results demonstrated clear A_s_‐dependent trends across multiple biological endpoints. Specifically, nanoparticles with higher A_s_ values generally exhibited prolonged blood circulation, increased AUC, reduced clearance rates, decreased liver accumulation, and lower phagocytic‐cell uptake (Figure S12). These findings suggest that nanoparticle asymmetry plays an important role in regulating systemic transport and immune recognition in vivo. Among the investigated formulations, ASN‐5 exhibited the most favorable long circulation and immune evasion, mechanistically due to its altered hydrodynamic behavior and cell‐particle interactions upon anisotropy increase.

To further investigate the relationship between the in vitro dynamic cellular interaction results and the in vivo biological fate of ASNs, correlation analyses were performed between nanoparticle uptake under dynamic flow conditions and representative pharmacokinetic and biodistribution parameters. The results demonstrated that nanoparticles exhibiting lower cellular interaction/uptake under dynamic conditions generally showed prolonged blood circulation, reduced clearance rates, decreased liver accumulation, and lower Kupffer‐cell and splenic macrophage uptake in vivo (Figure S13). In addition, the newly added HUVEC‐based microfluidic flow experiments further supported the dynamic uptake observations. Under low‐shear laminar flow conditions, nanoparticles with higher asymmetry exhibited reduced endothelial cellular uptake and retention compared with more symmetric formulations. These results were also consistent with the CFD analyses, which predicted reduced wall interaction and altered hydrodynamic behavior for highly anisotropic nanoparticles under flow conditions. Collectively, these findings suggest that nanoparticle asymmetry influences flow‐mediated cellular interactions, which may subsequently affect RES recognition and organ‐specific biodistribution in vivo. Although the current in vitro models cannot fully replicate the complexity of physiological circulation and organ microenvironments, the combined in vitro, microfluidic, CFD, and in vivo analyses provide mechanistic support linking anisotropy‐dependent cellular interactions with biological fate in vivo.

## 3 Conclusions

This study establishes nanoscale anisotropy as a fundamental determinant of nanocarrier transport, biodistribution, and clearance in vivo by engineering silica nanocapsules with precisely tunable asymmetry. Systematic in vitro and in vivo analyses revealed that increasing anisotropy markedly suppressed cellular uptake under physiological shear flow, reduced hepatic sequestration, and extended systemic circulation. CFD simulations further confirmed that increasing nanoscale anisotropy suppresses flow‐mediated margination and reduces the near‐wall residence of nanocapsules within vascular networks. Mechanistic investigations further showed that anisotropic particles evade multiple clearance pathways by minimizing adhesion and phagocytic internalization across Kupffer cells, splenic macrophages, and circulating monocytes. Collectively, these findings highlight that the coupling between nanocarrier geometry and physiological flow governs biological fate—a principle long exploited in nature but underutilized in nanomedicine. This work underscores anisotropy engineering as a practical and powerful strategy to design long‐circulating, immune‐evasive nanocarriers with improved targeting and therapeutic performance.

## 4 Experimental Section

### 4.1 Materials

Tetraethyl orthosilicate (TEOS, 98%), (3‐aminopropyl) triethoxysilane (APTES), hexadecane (98%), dichloromethane (CH_2_Cl_2_, 99.5%) and cetyltrimethylammonium chloride (CTAC, 99%) were purchased from Aladdin Biochemical Technology Co., Ltd. (Shanghai, China). Lutensol AT50 was supplied by BASF SE. (Ludwigshafen, Germany). Cyanine5 NHS Ester (Cy5‐SE) was purchased from MedChemExpress Co., Ltd. (New Jersey, USA). Hank's balanced salt solution (HBSS), and D‐Hank's balanced salt solution (D‐HBSS) were procured from Solarbio Science & Technology Co., Ltd. (Beijing, China). Cell Counting Kit‐8 (CCK‐8), 4',6‐diamidino‐2‐phenylindole (DAPI) and Red Blood Cell Lysis Buffer were obtained from Beyotime Biotechnology (Shanghai, China). Dulbecco's Modified Eagle Medium (DMEM), Roswell Park Memorial Institute (RPMI) 1640 medium, fetal bovine serum (FBS), and trypsin‐ethylene diamine tetraacetic acid (EDTA, 0.25%) were acquired from Thermo Fisher Bio‐Chemicals Co., Ltd. (Beijing, China). PE anti‐mouse CD146 antibody (168403), FITC anti‐mouse F4/80 antibody (123108), PE anti‐mouse CD11C antibody (117307), FITC anti‐mouse CD3 antibody (100203), FITC anti‐mouse CD19 antibody (115505), FITC anti‐mouse/human CD11b antibody (101205), PE anti‐mouse Ly‐6C antibody (128007), PerCP/Cyanine5.5 anti‐mouse Ly‐6G antibody (127615) and anti‐mouse CD16/32 antibody (156603) were obtained from BioLegend Inc. (California, USA). All solvents used in this study are analytical grade.

### 4.2 Synthesis of Anisotropic Silica Nanocapsules (ASNs)

Six ASNs with varying asymmetry (A_s_) from 1.0 to 1.8 were synthesized by using Sol‐Gel method following established procedures [15]. ASNs were synthesized in oil‐in‐water miniemulsions using the surface of oil nanodroplets as a template for the hydrolysis and condensation of alkoxysilanes. Specifically, 2.0 g (9.6 mmol) TEOS was first mixed with 125 mg hexadecane and 1 g dichloromethane to form the oil phase. Next, 30 mL of cationic surfactant CTAC aqueous solution (0.77 mg mL^−1^) was poured into the oil solution under stirring at 1000 rpm to pre‐emulsify for 10 min. The emulsion was then injected in a microfluidizer (Nano, Noozle Fluid Technology Co., Ltd., Shanghai, China) cooled with ice, or in ultrasonicator (JY92‐IIN, Ningbo Xinzhi Biotechnology Co., Ltd., Ningbo, China). The microfluidizer method operated at 60 MPa‐120 MPa and recirculated into the inlet reservoir for multiple cycles of processing to achieve uniform droplet size distribution. After microfluidization or ultrasonication, the emulsions were reacted at 25°C for 12 h to allow complete hydrolysis and condensation of TEOS, forming ASNs. The resulting nanoparticles were collected by centrifugation (1760 g, 10 min), washed three times with ethanol and deionized water, and finally redispersed or freeze‐dried for further characterization. To prepare nanocapsules with similar size but different anisotropy, operating pressure (30‐120 MPa) and cycles (3‐9) were designed and adjusted. The resulting miniemulsions were stirred at 1000 rpm for 12 h at 25°C to allow the hydrolysis and condensation of the silica precursor and the anisotropic growth of nanoparticles.

For fluorescent probe labeled ASNs, Cy5‐NHS ester was first coupled with APTES at a molar ratio of 1:1.1 to obtain fluorescently labeled silica precursors. The APTES‐Cy5 conjugates were then mixed with TEOS as the silica source. The proportion of APTES‐Cy5 in the total silane precursor was kept the same for all formulations. Before cellular and in vivo experiments, fluorescence intensity was measured at equal particle mass to confirm comparable labeling among the different formulations.

PEGylated ASNs were synthesized by exchanging the templating surfactant CTAC with the nonionic surfactant Lutensol AT50. PEGylated ASNs were denoted as ASN‐PEG. Specifically, 30 mg of Lutensol AT50 was added to 2 mL of ASNs dispersion. The dispersion was stirred at 1000 rpm for 2 h and then dialyzed against water with a dialysis bag (MWCO = 1000 kDa). Afterward, the dialyzed dispersion was centrifuged at 1760 g to remove the excess of Lutensol AT50. The pellet was redispersed in water and the dispersion was stirred at 1000 rpm for 24 h. All nanoparticle formulations used for in vivo pharmacokinetic, biodistribution, and cellular uptake analyses were prepared using this same Lutensol AT50‐based surfactant‐exchange protocol before administration. The obtained ASNs were finally collected and stored at 4°C for further characterization and biological experiments.

### 4.3 Characterization of ASNs

The hydrodynamic diameter (*D*_h_), polydispersity index (PDI) and zeta (*ζ*) potential of ASNs were measured by Malvern Zetasizer (Nano‐ZS ZEN3600, Malvern Instruments Co., Ltd., UK). Morphology of ASNs was characterized by using transmission electron microscopy (TEM, JEM‐2100EX, JEOL Ltd., Japan). The degree of Cy5 dye labeling was assessed using fluorescence spectroscopy (FLS1000, Edinburgh Instruments, Scotland, UK).

### 4.4 Suspension Stability Assay of ASNs

To evaluate sedimentation behavior under cell culture conditions, Cy5‐ASNs were dispersed in phenol red–free complete RPMI medium containing 10% FBS at a concentration of 200 µg mL^−1^. Samples were sonicated for 10 s prior to incubation. The initial fluorescence intensity (F_0_) was determined by measuring 10 µL of suspension (Ex = 649 nm, Em = 670 nm). Samples were then incubated under static conditions, and at indicated time points, 10 µL aliquots were withdrawn from one‐tenth of the liquid height below the meniscus to determine the fluorescence intensity (F_t_). The sedimentation index was calculated as (F_t_/F_0_) × 100%.

### 4.5 Cell Culture

Mouse monocyte macrophage leukaemia cell line (RAW264.7), human umbilical vein endothelial cells (HUVECs), and human monocytic leukemia cells (THP‐1) were obtained from the Type Culture Collection Committee of the Chinese Academy of Sciences. The cell lines were verified through morphological assessment and PCR‐based approaches to confirm their identities and ensure they were free of mycoplasma contamination. The cells were cultured in DMEM or RPMI 1640 supplemented with 10% FBS, 100 µg mL^−1^ streptomycin, and 100 U mL^−1^ penicillin.

### 4.6 Preliminary Safety Evaluation of ASNs

Cytotoxicity of the ASNs was assessed using the CCK8 assay. Three cell types (RAW264.7, HUVEC, and L929) were seeded in 96‐well plates at a density of 1 × 10^4^ cells per well. After 24 h, complete medium containing ASNs at different concentrations (0‐200 µg mL^−1^) were added to replace the old medium, followed by another 24 h of incubation. Next, each well received 100 µL of fresh medium and 10 µL of CCK8 reagent, and further incubated for 2 h. Finally, the absorbance of each well was measured at 450 nm with a microplate reader. Cell viability (%) was calculated using the formula: Cell viability (%) = [(A_e_‐A_b_)/(A_c_‐A_b_)] × 100%, where A_e_ is the absorbance of the experimental well, A_b_ is the absorbance of the blank well, and A_c_ is the absorbance of the control well.

### 4.7 Cellular Uptake Behaviors of ASNs

To investigate the cellular uptake behavior and internalization efficiency of ASNs, cellular uptake of Cy5‐labeled ASNs was examined in RAW 264.7 and THP 1 cells using flow cytometry (FCM, Beckman Moflo XDP, Beckman Coulter, Germany). Cells were seeded in 6‐well plates at a density of 2.0 × 10^5^ cells and incubated for 24 h. Next, cells were treated with Cy5‐ASNs for 1 h. Afterwards, the cells were washed with PBS and the fluorescence intensity of the cells was measured by flow cytometry. Identically, images of RAW 264.7 or THP 1 cells subjected to the same dosing schedule as flow cytometry were captured using a confocal laser scanning microscope (Leica Co., Germany).

To simulate the dynamic flow process in blood vessels in vivo, HUVEC cells were cultured in 6‐well plates at 37°C and 5% CO_2_ until the cells covered the bottom of the plate. 2 mL of the nanoparticles in culture medium solution was added to each well. The 6‐well plates were then placed on an air shaker at 37°C. After shaking at different speeds for 1 h, the shaking speeds of 100 and 150 rpm corresponded to estimated shear stresses of approximately 0.15–0.35 Pa and 0.40–0.90 Pa, respectively. These values were estimated based on the culture‐plate geometry, medium volume, viscosity, orbital radius, and rotation speed, according to previously reported hydrodynamic analyses of orbital culture systems. The cells were washed with PBS and collected. The fluorescence intensity of the cells was measured by flow cytometry.

### 4.8 Animals

Female BALB/c mice (6 weeks old, 16–18 g) and male Sprague‐Dawley rats (6 weeks old, 210 ± 10 g) were purchased from Pengyue Experimental Animal Breeding Co. Ltd. (Jinan, China). The animals were maintained on a standard diet and water ad libitum at 22°C and 50%–60% relative humidity. All animal experiments were performed in accordance with the Institutional Animal Care and Use guidelines and the National Research Council's Guide. Animal experiments were approved by the Ethics and Animal Welfare Committee of the Ocean University of China (Application No. OUC‐SMP‐2024‐12‐07, OUC‐SMP‐2025‐02‐06).

### 4.9 Pharmacokinetic Study of ASNs

To investigate the pharmacokinetic profiles of Cy5‐ASNs, SD rats were intravenously administered with various asymmetric ASNs (15 mg ASNs per kg). The rats were randomly divided into six groups (*n* = 4) spherical ASNs (SP) and five groups of anisotropic ASNs (ASN‐1–ASN‐5) with increasing degrees of shape anisotropy. Fluorescence intensity values were measured using a microplate reader (TECAN, Switzerland) before injection to ensure consistency. Blood samples were collected at various time points (0 min, 5 min, 15 min, 30 min, 1 h, 2 h, 4 h, 8 h, 12 h, and 24 h) post‐administration, and plasma was obtained by centrifugation. The fluorescence intensity was measured using a microplate reader (E_x_ = 640 nm, E_m_ = 685 nm), and the plasma concentrations of Cy5‐ASNs at different time points were calculated based on the plasma standard curve of ASNs

### 4.10 In Vivo Biodistribution Imaging of ASNs

To evaluate the in vivo biodistribution and organ accumulation of ASNs, BALB/c mice were intravenously injected with 0.2 mL of Cy5‐labeled ASNs. After 1 h, the mice were euthanized and their major organs (heart, liver, spleen, lung, and kidney) were collected for biodistribution analysis using the IVIS Spectrum CT imager (PerkinElmer, Waltham, USA) and their fluorescence intensities were recorded.

### 4.11 Confocal Imaging of ASNs Under Microfluidic Shear Flow

HUVECs were seeded into µ‐Slide VI 0.4 flow chambers (ibidi, Cat. No. 80606), which contain six independent microchannels, and cultured at 37°C in a humidified atmosphere containing 5% CO_2_ until reaching 80%–90% confluence. Specifically, the six microchannels were divided into two groups: three channels were connected to a peristaltic pump and used as the flow group, whereas the remaining three channels were not connected to the pump and served as the static group. In each group, SP, ASN‐3, and ASN‐5 were separately added to one independent microchannel. Cy5‐labeled ASNs were dispersed in complete culture medium at a concentration of 200 µg mL^−^^1^. For the static group, nanoparticle‐containing medium was added to the channels and maintained without flow. For the flow group, nanoparticle‐containing medium was continuously perfused through the microchannels for 1 h using a peristaltic pump (LSP04‐1Y, Longer Precision Pump Co., Ltd., China), generating a wall shear stress of 1 dyn cm^−^^2^. During this process, fresh nanoparticle‐containing medium was continuously introduced from the inlet and discharged from the outlet, thereby maintaining unidirectional flow throughout the treatment period. During this 1 h treatment, the flow group was maintained under continuous pump‐driven flow, whereas the static group was maintained without pump connection. Following treatment, the channels were washed three times with phosphate‐buffered saline (PBS) to remove unbound nanoparticles. Cells were fixed with 4% paraformaldehyde for 15 min and stained with DAPI for 10 min to visualize nuclei. The cellular uptake and intracellular distribution of nanoparticles were observed using a confocal laser scanning microscope (CLSM, Leica Microsystems, Germany). Cy5 fluorescence was used to visualize nanoparticles, while DAPI fluorescence was used to identify cell nuclei. All images were acquired using identical imaging settings and processed with LAS X software.

### 4.12 Computational Fluid Dynamics (CFD) Simulation of ASNs in Blood Vessels

To systematically analyze the transport behavior of ASNs and their interactions with the vascular wall under flow conditions, we employed a unidirectionally coupled computational fluid dynamics–discrete element method (CFD–DEM) framework to simulate nanoparticles with varying aspect ratios in hepatic sinusoidal vessels. A CFD model was first established to describe blood flow in hepatic sinusoidal vessels. The flow domain was idealized as a snake‐shaped channel with a slightly flattened elliptical cross‐section and discretized using tetrahedral elements (95 026 elements in total). Mesh quality was evaluated using the skewness criterion, with all elements exhibiting skewness values below 0.8. Given the extremely low Reynolds number in hepatic sinusoids (Re ≪ 1), the flow was assumed to be laminar. Blood properties were assigned according to reported values for hepatic sinusoids, with a density of approximately 1050 kg·m^−^^3^ and a dynamic viscosity of 4.0 × 10^−^^3^ Pa·s [39, 40]. A uniform inlet velocity of 350 µm·s^−^^1^ was prescribed at the channel inlet [41], while a pressure‐outlet boundary condition was applied at the outlet.

A DEM model was subsequently constructed to simulate nanoparticle dynamics. Four types of ASNs were considered, including spherical particles (SP) and three anisotropic nanoparticles (AS1, AS3, and AS5) with increasing asymmetry ratios of approximately 1.21, 1.49, and 1.76, respectively. ASNs were assigned a uniform density of 2200 kg·m^−^^3^ [42]. For numerical stability and computational efficiency, particle stiffness was reduced to 0.5 GPa, consistent with common DEM practices [43]. A unidirectional CFD–DEM coupling scheme was employed, in which the fluid velocity field drove particle motion via hydrodynamic drag. Nanoparticles were injected at the inlet with random initial positions and orientations. In total, 13 211 particles were released, with approximately equal numbers for each particle type.

### 4.13 Liver Clearance Mechanism of ASNs

To analyze the hepatic clearance mechanism of ASNs, liver tissues were harvested from mice 1 h post‐injection, and hepatic cell populations were isolated using a two‐step collagenase perfusion method based on previous reports [44]. Briefly, mice were anesthetized with isoflurane, and the abdominal cavity was surgically exposed. The abdominal aorta was cannulated to circulate pre‐warmed HBSS (lacking Ca^2+^ and Mg^2+^ and containing 0.5 × 10^−3^ M EDTA and 25 × 10^−3^ M HEPES). The hepatic portal vein was cut off to enable the remaining blood to be flushed from the liver for 5 min. Liver digestion buffer (100 CDU mL^−1^ collagenase type IV and 25 × 10^−3^ M HEPES in HBSS) was then circulated for 5 min to digest the liver. Then the digested liver was collected and incubated in 10 mL of liver digestion buffer at 37°C for 30 min. Ice‐cold PBS solution (10 mL) was added to the digesting liver to end the digestion. The mixture was centrifugated at 50 g for 3 min at 4°C to separate the hepatocyte‐enriched fraction in the pellet from nonparenchymal cells in the supernatant. The hepatocyte‐enriched fraction was analyzed as HCs for downstream quantification. After staining Kupffer cells and LSECs with FITC F4/80 and PE CD146, respectively, the FCM was performed to analyze the uptake of ASNs in different liver cells.

### 4.14 Spleen Clearance Mechanism of ASNs

To evaluate the cellular uptake of ASNs by different immune cell populations in the spleen, after the liver is perfused, remove the spleen. A single‐cell suspension was prepared from the spleen by mechanical dissociation through a 70 µm mesh with gentle grinding. The cells were then washed in PBS and collected by centrifugation at 400 g for 5 min. Red blood cell lysis buffer was then added to the obtained cells, and immune cells were obtained after centrifugation at 400 g and 4°C for 5 min. After staining with the corresponding antibodies (anti‐mouse CD3 antibody for T cells, anti‐mouse CD19 antibody for B cells, anti‐mouse CD11c antibody for dendritic cells and anti‐mouse F4/80 antibody for Macrophages), the uptake behaviors of ASNs in different immune cells were analyzed by using FCM.

### 4.15 Blood Clearance Mechanism of ASNs

To analyze the blood clearance behaviors of ASNs, BALB/c mice were intravenously injected with 0.6 mL of Cy5‐ASNs (30 mg ASNs per kg body weight), including SP and ASNs with different A_s_ (ASN‐1‐ASN‐5). Blood samples were collected at 1 h post‐injection of ASNs and centrifugated at 2751 g for 10 min at 4°C to obtain the cells. Red blood cell lysis buffer was then added to the obtained cells, and leukocytes were obtained after centrifugation at 300 g and 4°C for 5 min. After staining with the corresponding antibodies (anti‐mouse CD11b antibody and anti‐mouse Ly‐6G antibody for neutrophils, anti‐mouse CD11b antibody and Ly‐6C antibody for monocytes, anti‐mouse CD3 antibody for T cells, anti‐mouse CD19 antibody for B cells and anti‐mouse CD11c antibody for dendritic cells), the uptake behaviors of ASNs in different immune cells were analyzed by using FCM.

### 4.16 Calculation Method for Contribution Rate

The relative contribution rate was calculated as follows: The total NPs uptake by a given cell population in a given organ is approximated as the sum of the values obtained by multiplying the MFI and the cell number:

$$\mathrm{Total}\;\mathrm{uptak}e_{i}=\mathrm{MF}I_{i}\;\times \;N_{i}$$

where MFI_i_ represents the MFI measured by flow cytometry for cell population i, and N_i_ represents the cell number in that cell population.

The total NPs uptake across all analyzed cell populations in a given organ is then calculated as below:

$$\mathrm{Total}\;\mathrm{uptak}e_{\mathrm{organ}}=\sum_{i}\left(\mathrm{MF}I_{i}\times \;N_{i}\right)$$

where j represents all the cell populations analyzed in a given organ.

Then, the contribution rate of the given cell population i to organ‐associated NP clearance is therefore calculated as below:

$$\;\mathrm{Contribution}\;\mathrm{rat}e_{i}=\frac{\mathrm{MF}I_{i}\;\times \;N_{i}}{\sum_{i}\mathrm{MF}I_{i}\;\times \;N_{i}}$$

Since the cell number for a given cell population (N_i_) can be expressed as the product of the total cell counts in the organ and its fractional abundance (N_i_ = N_total_ × Fraction_i_), the formulation can be simplified to:

$$\mathrm{Contributio}n_{i}=\frac{\mathrm{MF}I_{i}\;\times \;\mathrm{Fractio}n_{i}}{\sum_{i}\mathrm{MF}I_{i}\;\times \;\mathrm{Fractio}n_{i}}$$

Here, MFI_i_ is the background‐corrected MFI for cell population i, and Fraction_i_ is its relative cell abundance within the given organ. The denominator represents the overall cell populations analyzed, normalizing the total contribution to 100%. The cell population fractions used in this formulation were broadly used in previously published, well‐established studies reporting the relative abundance of major immune and parenchymal cell populations in mouse liver, spleen, and blood [45, 46, 47, 48].

### 4.17 Statistical Analysis

Background fluorescence and autofluorescence from untreated control samples were subtracted where applicable. For flow cytometry analysis, debris, doublets, and dead cells were excluded prior to data analysis. For cell‐type contribution analysis, background‐subtracted mean fluorescence intensity (MFI) values were used. All experiments were performed with at least three independent replicates (*n* ≥ 3), and data are presented as mean ± standard deviation (mean ± SD). Statistical analyses were conducted using GraphPad Prism 9. For comparisons among multiple groups, one‐way ANOVA followed by Tukey's multiple‐comparison test was used unless otherwise indicated, with p < 0.05 considered statistically significant. *, **, ***, and **** represent *p* < 0.05, *p* < 0.01, *p* < 0.001, and p < 0.0001, respectively.

## Author Contributions

**Xiaofei Li**: conceptualization, investigation, Writing – original draft, Writing – review and editing, methodology, software, formal analysis, project administration, data curation, supervision.Yingjie Yu: conceptualization, investigation, writing – original draft, writing – review and editing, methodology. Tianchang He: conceptualization, investigation, writing – original draft, methodology, writing – review and editing. Xiaoyan Fang: methodology, writing – review and editing, writing – original draft. Wenhua Yang: writing – original draft, writing – review and editing, methodology. Luying Zhou: writing – original draft, writing – review and editing, methodology. Hao Fan: methodology, software. Guimei Lin: methodology. Daniel Crespy: writing – review and editing, supervision. **Katharina Landfester**: writing – review and editing, supervision. **Junfeng Zhang**: funding acquisition, conceptualization.Shuai Jiang: conceptualization, funding acquisition, writing – review and editing, supervision, investigation, resources.

## Conflicts of Interest

The authors declare no conflicts of interest.

## Supporting information




**Supporting File 1**: advs77000‐sup‐0001‐SuppMat.docx.

**Supporting File 2**: advs77000‐sup‐0002‐VideoS1.mp4.

## Data Availability

The data that support the findings of this study are available from the corresponding author upon reasonable request.

## References

[1] H. Almeida , G. Traverso , B. Sarmento , and J. das Neves , “Nanoscale Anisotropy for Biomedical Applications,” Nature Reviews Bioengineering 2, no. 7 (2024): 609–625, 10.1038/s44222-024-00169-2.

[2] E. Ben‐Akiva , R. A. Meyer , H. Yu , J. T. Smith , D. M. Pardoll , and J. J. Green , “Biomimetic Anisotropic Polymeric Nanoparticles Coated with Red Blood Cell Membranes for Enhanced Circulation and Toxin Removal,” Science Advances 6, no. 16 (2020): aay9035, 10.1126/sciadv.aay9035. PMC7239698 32490199

[3] V. Kudryavtseva and G. B. Sukhorukov , “Features of Anisotropic Drug Delivery Systems,” Advanced Materials 36, no. 14 (2024): 2307675, 10.1002/adma.202307675. 38158786

[4] J. T. Lovegrove , B. Kent , S. Förster , C. J. Garvey , and M. H. Stenzel , “The Flow of Anisotropic Nanoparticles in Solution and in Blood,” Exploration 3, no. 6 (2023): 220075, 10.1002/EXP.20220075. PMC10742203 38264690

[5] M. A. Constantino , M. Jabbarzadeh , H. C. Fu , and R. Bansil , “Helical and Rod‐Shaped Bacteria Swim in Helical Trajectories with Little Additional Propulsion from Helical Shape,” Science Advances 2, no. 11 (2016): 1601661, 10.1126/sciadv.1601661. PMC5262464 28138539

[6] L. Mesarec , W. Góźdź , A. Iglic , V. Kralj‐Iglic , E. G. Virga , and S. Kralj , “Normal Red Blood Cells′ Shape Stabilized by Membrane's in‐Plane Ordering,” Scientific Reports 9, no. 1 (2019): 19742, 10.1038/s41598-019-56128-0. 31875042 PMC6930264

[7] K. Hayashi , S. Yamada , W. Sakamoto , E. Usugi , M. Watanabe , and T. Yogo , “Red Blood Cell‐Shaped Microparticles with a Red Blood Cell Membrane Demonstrate Prolonged Circulation Time in Blood,” ACS Biomaterials Science & Engineering 4, no. 8 (2018): 2729–2732, 10.1021/acsbiomaterials.8b00197. 33434998

[8] W. Yu , R. Liu , Y. Zhou , and H. Gao , “Size‐Tunable Strategies for a Tumor Targeted Drug Delivery System,” ACS Central Science 6, no. 2 (2020): 100–116, 10.1021/acscentsci.9b01139. 32123729 PMC7047275

[9] D. Zhang , M. J. Quayle , G. Petersson , J. R. van Ommen , and S. Folestad , “Atomic Scale Surface Engineering of Micro‐ to Nano‐sized Pharmaceutical Particles for Drug Delivery Applications,” Nanoscale 9, no. 32 (2017): 11410–11417, 10.1039/C7NR03261G. 28678265

[10] X. Huang , L. Li , T. Liu , et al., “The Shape Effect of Mesoporous Silica Nanoparticles on Biodistribution, Clearance, and Biocompatibility in Vivo,” ACS Nano 5, no. 7 (2011): 5390–5399, 10.1021/nn200365a. 21634407

[11] Y. Ma , K. Lan , B. Xu , et al., “Streamlined Mesoporous Silica Nanoparticles with Tunable Curvature from Interfacial Dynamic‐Migration Strategy for Nanomotors,” Nano Letters 21, no. 14 (2021): 6071–6079, 10.1021/acs.nanolett.1c01404. 34269590

[12] S. Shaw , G. C. Shit , and D. Tripathi , “Impact of Drug Carrier Shape, Size, Porosity and Blood Rheology on Magnetic Nanoparticle‐based Drug Delivery in a Microvessel,” Colloids and Surfaces A: Physicochemical and Engineering Aspects 639 (2022): 128370, 10.1016/j.colsurfa.2022.128370.

[13] Y. Sakamoto , S. Fujii , S. Takano , et al., “Manipulation of Macrophage Uptake by Controlling the Aspect Ratio of Graft Copolymer Micelles,” Nano Letters 24, no. 19 (2024): 5838–5846, 10.1021/acs.nanolett.4c01054. 38661003

[14] M. S. Alkanawati , F. R. Wurm , H. Thérien‐Aubin , and K. Landfester , “Large‐Scale Preparation of Polymer Nanocarriers by High‐Pressure Microfluidization,” Macromolecular Materials and Engineering 303, no. 1 (2018): 1700505, 10.1002/mame.201700505.

[15] S. Jiang , A. Kaltbeitzel , M. Hu , O. Suraeva , D. Crespy , and K. Landfester , “One‐Step Preparation of Fuel‐Containing Anisotropic Nanocapsules with Stimuli‐Regulated Propulsion,” ACS Nano 14, no. 1 (2020): 498–508, 10.1021/acsnano.9b06408. 31887001

[16] C. Horejs , “From Lipids to Lipid Nanoparticles to mRNA Vaccines,” Nature Reviews Materials 6, no. 12 (2021): 1075–1076, 10.1038/s41578-021-00379-9. 34567796 PMC8454296

[17] W. Poon , Y. N. Zhang , B. Ouyang , et al., “Elimination Pathways of Nanoparticles,” ACS Nano 13, no. 5 (2019): 5785–5798, 10.1021/acsnano.9b01383. 30990673

[18] C. I. Colino , J. M. Lanao , and C. Gutierrez‐Millan , “Targeting of Hepatic Macrophages by Therapeutic Nanoparticles,” Frontiers in Immunology 11 (2020): 00218, 10.3389/fimmu.2020.00218. PMC7065596 32194546

[19] K. Jiang , K. Tian , Y. Yu , et al., “Kupffer Cells Determine Intrahepatic Traffic of PEGylated Liposomal Doxorubicin,” Nature Communications 15, no. 1 (2024): 6136, 10.1038/s41467-024-50568-7. PMC11271521 39033145

[20] A. Chow , B. D. Brown , and M. Merad , “Studying the Mononuclear Phagocyte System in the Molecular Age,” Nature Reviews Immunology 11, no. 11 (2011): 788–798, 10.1038/nri3087. 22025056

[21] J. Lu , X. Gao , S. Wang , et al., “Advanced Strategies to Evade the Mononuclear Phagocyte System Clearance of Nanomaterials,” Exploration 3, no. 1 (2023): 20220045, 10.1002/EXP.20220045. 37323617 PMC10191055

[22] S. Dasgupta , T. Auth , and G. Gompper , “Wrapping of Ellipsoidal Nano‐particles by Fluid Membranes,” Soft Matter 9, no. 22 (2013): 5473–5482, 10.1039/C3SM50351H.

[23] F. Villanueva‐Flores , A. Castro‐Lugo , O. T. Ramírez , and L. A. Palomares , “Understanding Cellular Interactions with Nanomaterials: Towards a Rational Design of Medical Nanodevices,” Nanotechnology 31, no. 13 (2020): 132002, 10.1088/1361-6528/ab5bc8. 31770746 PMC7105107

[24] C. M. Warboys , M. Ghim , and P. D. Weinberg , “Understanding Mechanobiology in Cultured Endothelium: A Review of the Orbital Shaker Method,” Atherosclerosis 285 (2019): 170–177, 10.1016/j.atherosclerosis.2019.04.210. 31096159 PMC6570700

[25] R. Driessen , F. Zhao , S. Hofmann , C. Bouten , C. Sahlgren , and O. Stassen , “Computational Characterization of the Dish‐In‐A‐Dish, a High Yield Culture Platform for Endothelial Shear Stress Studies on the Orbital Shaker,” Micromachines 11, no. 6 (2020): 552, 10.3390/mi11060552. 32486105 PMC7345652

[26] A. Fernandes , V. Hosseini , V. Vogel , and R. D. Lovchik , “Engineering Solutions for Biological Studies of Flow‐Exposed Endothelial Cells on Orbital Shakers,” PLoS ONE 17, no. 1 (2022): 0262044, 10.1371/journal.pone.0262044. PMC8782315 35061745

[27] Y. Y. Chen , A. M. Syed , P. MacMillan , J. V. Rocheleau , and W. C. W. Chan , “Flow Rate Affects Nanoparticle Uptake into Endothelial Cells,” Advanced Materials 32, no. 24 (2020): 201906274, 10.1002/adma.201906274. 32383233

[28] T. Iskratsch , H. Wolfenson , and M. P. Sheetz , “Appreciating Force and Shape — The Rise of Mechanotransduction in Cell Biology,” Nature Reviews Molecular Cell Biology 15, no. 12 (2014): 825–833, 10.1038/nrm3903. 25355507 PMC9339222

[29] T. J. Nawara , J. Yuan , L. D. Seeley , E. Sztul , and A. L. Mattheyses , “Fluidic Shear Stress Alters Clathrin Dynamics and Vesicle Formation in Endothelial Cells,” Biophysical Journal 124, no. 11 (2025): 1763–1770, 10.1016/j.bpj.2024.06.007. 38853434 PMC12256901

[30] M. Cooley , A. Sarode , M. Hoore , D. A. Fedosov , S. Mitragotri , and A. Sen Gupta , “Influence of Particle Size and Shape on Their Margination and Wall‐adhesion: Implications in Drug Delivery Vehicle Design across Nano‐to‐Micro Scale,” Nanoscale 10, no. 32 (2018): 15350–15364, 10.1039/C8NR04042G. 30080212 PMC6247903

[31] T. Zhao , R. Lin , B. Xu , et al., “Mesoporous Nano‐Badminton with Asymmetric Mass Distribution: How Nanoscale Architecture Affects the Blood Flow Dynamics,” Journal of the American Chemical Society 145, no. 39 (2023): 21454–21464, 10.1021/jacs.3c07097. 37726207

[32] K. M. Tsoi , S. A. MacParland , X.‐Z. Ma , et al., “Mechanism of Hard‐Nanomaterial Clearance by the Liver,” Nature Materials 15, no. 11 (2016): 1212–1221, 10.1038/nmat4718. 27525571 PMC5132626

[33] A. C. Anselmo , V. Gupta , B. J. Zern , et al., “Delivering Nanoparticles to Lungs While Avoiding Liver and Spleen through Adsorption on Red Blood Cells,” ACS Nano 7, no. 12 (2013): 11129–11137, 10.1021/nn404853z. 24182189 PMC4128963

[34] E. Blanco , H. Shen , and M. Ferrari , “Principles of Nanoparticle Design for Overcoming Biological Barriers to Drug Delivery,” Nature Biotechnology 33, no. 9 (2015): 941–951, 10.1038/nbt.3330. PMC4978509 26348965

[35] J. Li and H. Wang , “Selective Organ Targeting Nanoparticles: From Design to Clinical Translation,” Nanoscale Horizons 8, no. 9 (2023): 1155–1173, 10.1039/D3NH00145H. 37427677

[36] L. Wang , S. Quine , A. N. Frickenstein , et al., “Exploring and Analyzing the Systemic Delivery Barriers for Nanoparticles,” Advanced Functional Materials 34, no. 8 (2024): 202308446, 10.1002/adfm.202308446. PMC11142462 38828467

[37] H. H. Gustafson , D. Holt‐Casper , D. W. Grainger , and H. Ghandehari , “Nanoparticle Uptake: The Phagocyte Problem,” Nano Today 10, no. 4 (2015): 487–510, 10.1016/j.nantod.2015.06.006. 26640510 PMC4666556

[38] A. C. Wauters , J. F. Scheerstra , M. M. T. van Leent , et al., “Polymersomes with Splenic Avidity Target Red Pulp Myeloid Cells for Cancer Immunotherapy,” Nature Nanotechnology 19, no. 11 (2024): 1735–1744, 10.1038/s41565-024-01727-w. PMC11567884 39085390

[39] A. M. T. Rojas and S. Lorente , “1D‐Model of the Human Liver Circulatory System,” Computer Methods and Programs in Biomedicine 238 (2023): 107612, 10.1016/j.cmpb.2023.107612. 37224726

[40] V. Rezania , D. Coombe , and J. A. Tuszynski , “A Physiologically‐based Flow Network Model for Hepatic Drug Elimination III: 2D/3D DLA Lobule Models,” Theoretical Biology and Medical Modelling 13, no. 1 (2016): 9, 10.1186/s12976-016-0034-5. 26939615 PMC4778290

[41] J. Fan , C.‐J. Chen , Y.‐C. Wang , W. Quan , J.‐W. Wang , and W.‐G. Zhang , “Hemodynamic Changes in Hepatic Sinusoids of Hepatic Steatosis Mice,” World Journal of Gastroenterology 25, no. 11 (2019): 1355–1365, 10.3748/wjg.v25.i11.1355. 30918428 PMC6429340

[42] G. Yalçın , S. Öztuna , A. S. Dalkılıç , and S. Wongwises , “Effect of Particle Size on SiO_2_ Nanofluid Viscosity Determined by a Two‐Step Method,” Journal of Thermal Analysis and Calorimetry 149, no. 23 (2024): 13681–13696, 10.1007/s10973-024-13403-1.

[43] C. Wen , Y. Hou , and P. Fang , “Exploration of Elastic Modulus Reduction and Contact Parameters Optimization for Matching Original DEM Simulation Responses,” Chemical Engineering Science 306 (2025): 121220, 10.1016/j.ces.2025.121220.

[44] R. Mendoza , I. Banerjee , and S. C. Reghupaty , “Isolation and Culture of Mouse Hepatocytes and Kupffer Cells (KCs),” in Methods in Molecular Biology (2022), 2455, 10.1007/978-1-0716-2128-8_7. PMC8919380 35212987

[45] J. L. Baratta , A. Ngo , B. Lopez , N. Kasabwalla , K. J. Longmuir , and R. T. Robertson , “Cellular Organization of Normal Mouse Liver: A Histological, Quantitative Immunocytochemical, and Fine Structural Analysis,” Histochemistry and Cell Biology 131, no. 6 (2009): 713–726, 10.1007/s00418-009-0577-1. 19255771 PMC2761764

[46] E. Arlt , A. Kindermann , A.‐K. Fritsche , A. Navarrete Santos , H. Kielstein , and I. Bazwinsky‐Wutschke , “A Flow Cytometry‐Based Examination of the Mouse White Blood Cell Differential in the Context of Age and Sex,” Cells 13, no. 18 (2024): 1583, 10.3390/cells13181583. 39329764 PMC11430320

[47] J. A. Hensel , V. Khattar , R. Ashton , and S. Ponnazhagan , “Characterization of Immune Cell Subtypes in Three Commonly Used Mouse Strains Reveals Gender and Strain‐specific Variations,” Laboratory Investigation 99, no. 1 (2019): 93–106, 10.1038/s41374-018-0137-1. 30353130 PMC6524955

[48] T. He , L. Zhu , J. Ding , et al., “Macrophage Phenotype–Dependent Protein Corona Formation Governs Ligand Accessibility and Immune Clearance of Biomimetic Nanoparticles,” Small 22, no. 22 (2026): 14389, 10.1002/smll.202514389. PMC13089100 41718688

