# Supplementary material for: Engineering Morphological Anisotropy to Control the In Vivo Transport Dynamics, Clearance, and Biodistribution of Silica Nanocarriers
Source: Adv Sci (Weinh). 2026 Aug 5;13(60):e77000. doi: 10.1002/advs.77000 (PMC13440192; doi:10.1002/advs.77000)
Supplement: Supplementary file 1 [file ADVS-13-e77000-s002.docx]

Supporting Information

**Engineering Morphological Anisotropy to Control the *In Vivo* Transport Dynamics, Clearance, and Biodistribution of Silica Nanocarriers**

*Xiaofei Li,^+^ Yingjie Yu,^+^ Tianchang He,^+^ Xiaoyan Fang, Wenhua Yang, Luying Zhou, Hao Fan, Guimei Lin, Daniel Crespy, Katharina Landfester,* Junfeng Zhang,* Shuai Jiang**

X. Li, Y. Yu, T. He, X. Fang, W. Yang, L. Zhou, H. Fan, J. Zhang, S. Jiang

Key Laboratory of Marine Drugs, Chinese Ministry of Education, School of Medicine and Pharmacy, Ocean University of China, Qingdao 266003, China.

Laboratory for Marine Drugs and Bioproducts, Qingdao Marine Science and Technology Center, Qingdao 266237, China.

E-mail: [jfzhang@ouc.edu.cn](mailto:jfzhang@ouc.edu.cn); [jiangshuai@ouc.edu.cn](mailto:jiangshuai@ouc.edu.cn)

G. Lin

School of Pharmaceutical Science, Shandong University, Jinan 250012, P. R. China.

D. Crespy

Department of Materials Science and Engineering, School of Molecular Science and Engineering, Vidyasirimedhi Institute of Science and Technology (VISTEC), Rayong 21210, Thailand

K. Landfester

Max Planck Institute for Polymer Research, Ackermannweg 10, 55128 Mainz, Germany

E-mail: [landfester@mpip-mainz.mpg.de](mailto:landfester@mpip-mainz.mpg.de)

^[+]^ Xiaofei Li, Yingjie Yu, and Tianchang He contributed equally to this work.

**Table S1**. Pharmacokinetic model goodness-of-fit and diagnostic parameters obtained from PKSolver.

| Parameter | SP | ASN-1 | ASN-2 | ASN-3 | ASN-4 | ASN-5 |
| --- | --- | --- | --- | --- | --- | --- |
| Goodness-of-fit (R²) | 0.9960 | 0.9945 | 0.9938 | 0.9917 | 0.9917 | 0.9984 |
| WSS | 348.18 | 744.27 | 780.07 | 1185.82 | 1656.37 | 385.05 |
| SE | 8.34 | 12.20 | 12.49 | 15.40 | 18.20 | 8.78 |
| AIC | 60.67 | 67.51 | 67.93 | 71.70 | 74.71 | 61.58 |
| SC | 61.46 | 68.30 | 68.72 | 72.49 | 75.50 | 62.37 |

*Note*: R², coefficient of determination; WSS, weighted sum of squares; SE, standard error; AIC, Akaike information criterion; SC, Schwarz criterion. Pharmacokinetic parameters were calculated using a standard two-compartment model implemented in PKSolver.

**Table S2**. Estimated shear stress under orbital-shaker conditions.

| Rotation speed (rpm) | Estimated shear stress (Pa) | Representative physiological environment |
| --- | --- | --- |
| 100 | 0.15-0.35 | Hepatic sinusoids / capillaries |
| 150 | 0.40-0.90 | Capillaries / venules |

*Note*: Estimated shear stresses were calculated based on the culture-plate geometry, medium volume, medium viscosity, orbital radius, and rotation speed. Values are intended to provide physiological context and should not be interpreted as exact local shear stresses because orbital shaking generates spatially heterogeneous flow fields.

**Figure S1.** Microfluidization-enabled modulation of ASN anisotropy. TEM images of ASNs prepared from emulsions containing 96 wt% TEOS. Capsule anisotropy increases with microfluidization cycles (3–9 cycles, horizontal) and operating pressure (60–120 MPa, vertical).

**Figure S2.** Optical characterization of Cy5-labeled ASNs. (a) Absorption and (b) emission spectra of free Cy5 dye and Cy5-labeled ASNs.

**Figure S3.** Representative fluorescence images of ASNs in blood at 1 h post-injection.

**Figure S4.** Fluorescence labeling consistency of ASNs. (a) Absolute fluorescence intensity per 1 mg particles. (b) Relative fluorescence intensity of Cy5-labeled ASNs at an equal particle mass concentration of 10 mg mL⁻¹, normalized to SP. Data are presented as mean ± SD (*n* = 3). Statistical significance was analyzed using one-way ANOVA followed by Tukey’s multiple-comparison test.

**Figure S5.** Time-dependent suspension stability of Cy5-labeled ASNs in complete RPMI medium, evaluated by fluorescence-based sedimentation analysis. (*n* = 3).

**Figure S6.** Cellular uptake pathways of ASNs investigated using pharmacological inhibitors. (a) Relative uptake efficiency of ASNs after pretreatment with chlorpromazine (CPZ), 5-(N-ethyl-N-isopropyl) amiloride (EIPA), dynasore (Dyna), and nocodazole (Noco), normalized to untreated control. (b) Heatmap showing quantitative uptake under different inhibitory conditions (*n* = 3).

**Figure S7.** Confocal laser scanning microscopy images of HUVECs incubated with three ASNs (SP, ASN-3 and ASN-5) under static and dynamic (100 rpm) conditions. Scale bar: 25 *μ*m.

**Figure S8.** Geometry of the snake-shaped CFD channel with a slightly flattened elliptical cross-section.

**Figure S9.** Representative gating strategy applied for flow cytometric analysis of ASNs^+^ populations in three types of liver cells. The Hepatocyte-enriched cells and non-parenchymal cells were gated for mononuclear (both FSC-H vs. SSC-H) and singlet (SSC-A vs. SSC-H) subpopulations. The hepatocyte-enriched cell population was analyzed as HC for downstream quantification. Kupffer cells (KCs)were gated as the F4/80^+^ subpopulation and liver sinusoidal endothelial cells (LSECs) as the CD146^+^ subpopulation.

**Figure S10.** Representative gating strategy for flow cytometric analysis of ASNs^+^ populations in four types of spleen cells. Isolated cells were gated for selection of lymphocyte, monocyte (both FSC-H vs. SSC-H) and singlet (SSC-A vs. SSC-H) populations, and the following were gated further: CD19^+^CD3^-^for B cells (BCs), CD3^+^CD19^-^ for T cells (TCs), F4/80^+^ for macrophages (MACs), and CD11C^+^ for dendritic cells (DCs).

**Figure S11.** Representative gating strategy for flow cytometric analysis of ASNs^+^ populations in four types of blood cells. Isolated cells were gated for selection of lymphocyte, monocyte (both FSC-H vs. SSC-H) and singlet (SSC-A vs. SSC-H) populations, and the following were gated further: CD3^+^ for TCs, CD11C^+^ for DCs, CD11B^+^Ly6G^+^for neutrophils (Neus), and CD11B^+^Ly6C^+^for monocytes (Mons).

**Figure S12.** Correlation analysis between nanoparticle As and *in vivo* biological behaviors. Correlation plots showing the relationship between As and (a) circulation half-life (t₁/₂), (b) clearance rate, (c) liver accumulation, and (d) Kupffer cell uptake. Linear regression analyses were performed to evaluate the dependence of pharmacokinetic and biodistribution parameters on nanoparticle asymmetry. Data points represent individual biological replicates (*n* = 3). R² and p-values are indicated in each panel.

**Figure S13.** Correlation between cellular uptake in the *in vitro* microfluidic model and *in vivo* pharmacokinetics and biodistribution of ASNs. Correlation plots showing the relationship between HUVEC MFI under dynamic flow conditions (100 rpm) and (a) circulation half-life (t₁/₂), (b) clearance rate (CL), (c) liver accumulation, and (d) Kupffer cell uptake. Data points represent individual biological replicates (*n* = 3). R² and p-values are indicated in each panel.
